# Capillary Wave Driven Dynamics of Graphene Domains during Growth on Molten Metals

**DOI:** 10.1021/acs.jpclett.5c02321

**Published:** 2025-09-17

**Authors:** Kristýna Bukvišová, Radek Kalousek, Marek Patočka, Jakub Zlámal, Jakub Planer, Vojtěch Mahel, Daniel Citterberg, Libor Novák, Tomáš Šikola, Suneel Kodambaka, Miroslav Kolíbal

**Affiliations:** † CEITEC BUT, 48274 Brno University of Technology, Purkyňova 123, 612 00 Brno, Czech Republic; ‡ Thermo Fisher Scientific, Vlastimila Pecha 12, 627 00 Brno, Czech Republic; § Institute of Physical Engineering, 48274 Brno University of Technology, Technická 2, 616 69 Brno, Czech Republic; ∥ Department of Materials Science and Engineering, 1757 Virginia Polytechnic Institute and State University, Blacksburg, Virginia 24061, United States

## Abstract

Rheotaxy—growth of crystalline layers on molten surfaces—is considered as a promising approach for achieving large-scale monolayers of two-dimensional (2D) materials via seamless stitching of 2D domains during growth on molten metals. However, the mechanisms leading to this process are not well understood. Here, we present in situ microscopic observations of rheotaxy of graphene via chemical vapor deposition on molten gold and copper. We show that the graphene domains undergo translational and rotational motions, leading to self-assembly, during growth on molten metals. Using environmental and ultrahigh vacuum scanning electron microscopy and high-temperature (∼1300 K) atomic force microscopy, coupled with density functional theory and continuum modeling, we suggest that the observed graphene domain dynamics is due to forces arising from capillary waves on the surface of the liquid metal. Our results provide new insights into the mechanisms leading to self-assembly during rheotaxy of 2D layers.

## Graphical abstract

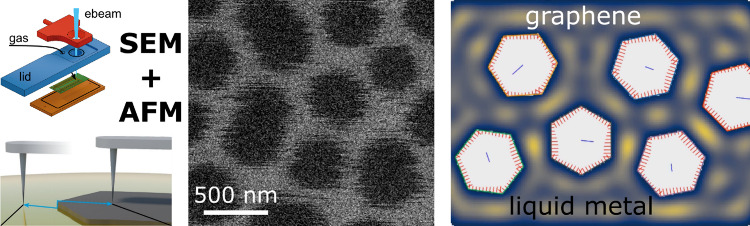

Since the discovery of graphene, considerable efforts have been aimed at the production of large-area graphene sheets.^1^ Among the several different synthesis approaches proposed to date, chemical vapor deposition (CVD) has been the most promising method for obtaining high-quality large-area layers of graphene.^2–6^ CVD onto polycrystalline foils and amorphous substrates typically yield polydomain graphene, i.e. with multiple rotational domains separated by boundaries; such graphene layers are undesirable for most applications. A prominent strategy to grow a large-scale single-domain graphene (and other 2D materials) has been to use single-crystalline substrates and by the optimal choice of growth parameters, large-domain and single-crystalline graphene layers have been obtained by stitching many unidirectionally aligned domains.^2–6^ Another successful approach has been to form a single nucleus on the substrate and let it grow into a single-domain layer under well-controlled growth conditions.^7^ Recently, rheotaxy—growth of crystalline thin films on molten surfaces^8–12^ —has been used to produce graphene layers with highly ordered domains via CVD on molten Cu.^13,14^ The absence of rigid substrate has been proposed^15^ to promote strain-free^16^ and seamless assembly of many grains that originated from numerous nucleation events during growth. As such, rheotaxy holds great promise even beyond the realm of graphene.^17–19^ The successful demonstration of large-area graphene growth via rheotaxy has motivated in situ studies, which have suggested that short-range electrostatic interactions and long-range capillary forces may be the factors contributing to self-assembly,^20–22^ however, the exact mechanisms are not known. Self-assembly has been observed previously in different nanoscale systems,^23^ being driven by e.g. strain relief in Stranski–Krastanov growth^24^ or electron density standing waves.^25^ At the mesocale, the long-range forces are required to arrange e.g. domains of 2D materials into regular patterns.^26^ Here, we focus on fundamental understanding of the dynamics of graphene domains on molten metals, with the aim of providing new insights into the mechanisms leading to self-assembly on liquid substrates.

We carried out graphene growth and monitoring experiments in situ in two different microscopes using ethylene as the carbon source. One set of experiments involved the growth of graphene layers on Au using a high-pressure (up to 150 Pa) MicroReactor^27,28^ and the other on Cu in an ultrahigh vacuum (UHV; base pressure ∼ 10^–7^ Pa) environment at temperatures (1073 K < *T* < 1390 K) above and below their melting points (*T*_m,Au_ = 1337.33 K and *T*_m,Cu_ = 1357.77 K)^29^ where the metals are either liquid or solid, respectively. The MicroReactor design (see ref 28 and also Methods in the Supporting Information, SI) enables simultaneous heating of the metal samples to high temperatures (*T* > *T*_m_) and dosing reactive gases at high pressures during the operation of SEM, which facilitates the observation of graphene nucleation and growth on solid and molten Au. The UHV experiments were carried out in a custom-designed SEM, in which we melted copper supported on a platinum wire that was used as a resistive heater, with the base pressure ∼ 10^–7^ Pa to ensure the cleanliness of the reactive molten surface. We chose copper because it is the most common metal catalyst for graphene CVD growth and was also used for graphene rheotaxy.^20,22,30^ Although gold is not a commonly used catalyst for graphene growth, we chose it due to its very low vapor pressure (∼10^–3^ Pa at *T* = 1200 K)^31^ and, more importantly, due to its inertness toward oxidation. We do not observe nucleation on the liquid surfaces in our experiments, presumably due to insufficient growth flux (even at the maximum permissible precursor pressures in our system), the absence of nucleation sites, and/or related requirement of very high supersaturation for nucleation. Therefore, we always nucleated graphene on solid substrate and continued the growth up to a certain domain size, after which the sample was rapidly molten. The precursor flow was kept constant and the SEM images acquired continuously (full movie documenting the experimental workflow is presented as Movie S1). All images and movies presented here were acquired using secondary electrons; beam conditions are stated in figure captions. We have performed several control experiments to assess the possible effect of the electron beam (momentum or charge transfer, local heating etc.) on the observed phenomena, concluding that the beam effects are negligible (see the SI for further details). Given that all our experiments are carried out on fairly large (tens of micrometers up to millimeter size) metal droplets and since all our detailed in situ observations are limited to fields of view much smaller than the substrate size, we expect that the influence of thermal gradients, if any, on graphene domain dynamics is insignificant. Further, we have found that the kinetics of nucleation, growth, and shape evolution of graphene domains depend on both the catalyst (e.g., Au vs Cu) and on its state (liquid vs solid), which will be discussed elsewhere. Using Raman spectroscopy (see the SI for more details), we have confirmed that the layers deposited on both solid and liquid Au and Cu surfaces in our in situ experiments are graphene.

Figure 1 shows a representative set of SEM images extracted from Movies S2 and S3 obtained during graphene growth on solid Au (Figure 1a) and then, after raising the temperature above the melting point *T*_m_, on liquid (Figure 1b). In these images, darker and lighter gray contrast features are individual graphene domains and metal surfaces, respectively. After melting Au substrate, we have instantly observed spontaneous assembly of the graphene domains into regular arrays, as documented in Figure 1b and Movie S3. (We have also observed similar phenomenon during graphene growth on molten Cu.) The domains assemble on the liquid within a few line scans of the electron beam. If compared to the very slow growth rate of graphene, the self-assembly can be treated as independent from the growth. Although the domains are supported on a molten metal, the centers of mass of floating graphene domains remain in-place (see Figure 1c), while the interdomain spacings *d* slowly shrink (Figure 1d). Intriguingly, the domains on the liquid surface (panel b) appear fuzzy in the in situ SEM images. Since the images are acquired by scanning with the electron beam, any change in the instantaneous positions of the domains within each frame results in blurred contours. Movies S3 and S4 reveal that the domains perform a complex motion, further termed wobbling, which includes both translational motion and rotations during growth. The wobbling of domains on the liquid metal is direct evidence that the domains are weakly bonded to molten metal surfaces during growth. We speculate (and justify below) that the free motion of the domains on the liquid surface leads to self-assembly.

**Figure 1:**
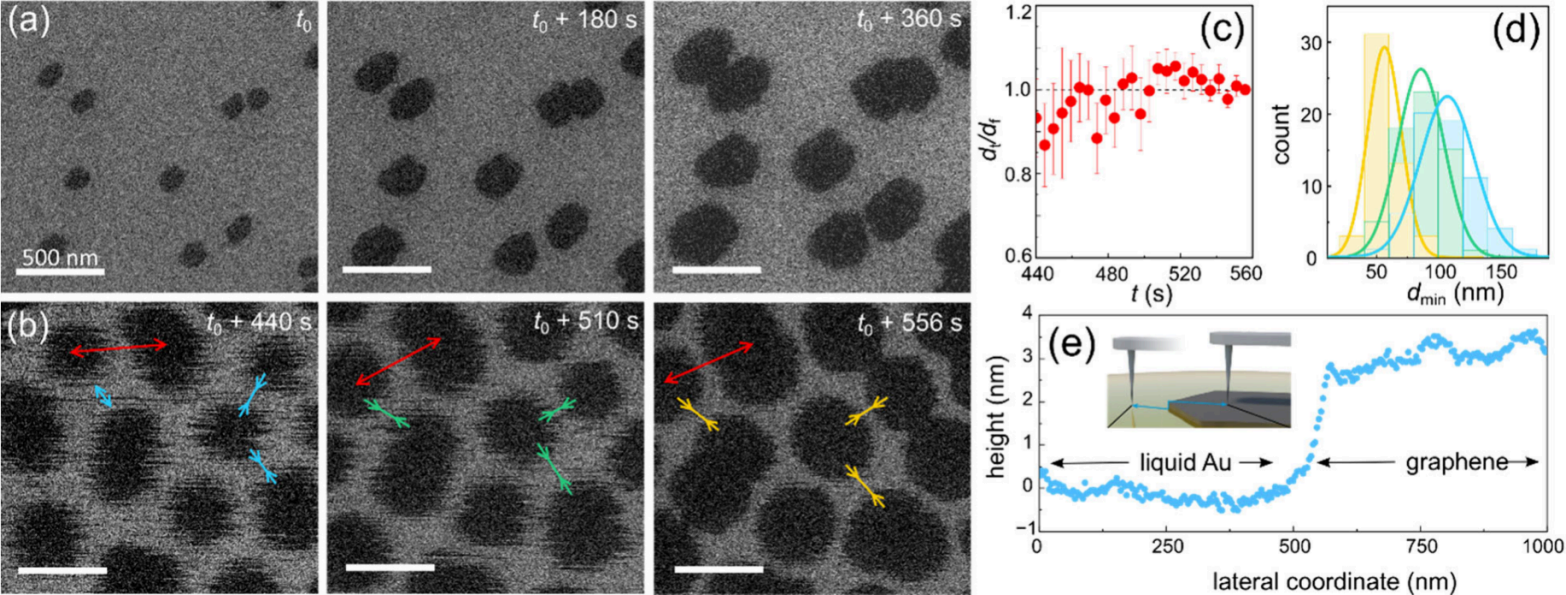
Graphene growth on solid Au and self-assembly on molten Au. Representative in situ scanning electron microscopy (SEM) images (extracted from Movies S2 and S3 in the SI, respectively) acquired (a) from a solid Au sample at temperature *T* = 1223 K and (b) after melting the same sample at *T* = 1373 K as a function of time *t* during exposure to the ethylene (C_2_H_4_) gas at 30 Pa. Time *t*_0_ marks the first frame in the image sequence. In the SEM images, graphene domains and Au substrate appear in a darker and lighter gray contrast, respectively. All of the scale bars are 500 nm. (c) Plot of the distances *d*_*t*_/*d*_f_ vs *t*, where *d*_*t*_ and *d*_f_ are defined as the distances, respectively, between centers-of-mass of neighboring domains at times *t* (highlighted by red arrows in panel b) and at *t*_f_ = *t*_0_ + 560 s. (d) Histograms of minimum distances *d*_min_ between adjacent domains, indicated by arrows in panel b with cyan, green, and yellow colors in the plot, respectively. (e) In situ high-temperature AFM measured topography profile of a floating graphene domain on molten Au, measured at 1343 K in high vacuum (10^–4^ Pa). We chose a graphene domain that was pinned to other domains and, hence, stable during the measurement. The inset shows a schematic of the measurement geometry; the topography map is shown in the SI.

Often, surface dynamical phenomena such as self-assembly, Ostwald ripening, etc. are attributed to capillary forces, which give rise to meniscus on liquid surfaces. For graphene floating on the molten metal, a liquid meniscus, if present, would be manifested by a rim around the individual domains in secondary electron images. On fully molten metals, the individual graphene domains and the surrounding liquid appear flat, homogeneous and rim-free in SEM images for both gold and copper. In order to further verify this conclusion, we used HT-AFM operated up to *T* ∼ 1380 K. Figure 1e shows an AFM topography line profile measured across the edge of a graphene domain floating on molten gold. Importantly, the liquid does not exhibit a meniscus (resolvable with our HT-AFM), in agreement with the SEM images (see detailed discussion in the SI).

We now focus on understanding the phenomenon of wobbling of graphene domains seen in SEM (Figure 1b, Movie S3). Figure 2a shows typical SEM images (extracted from Movie S5) of an individual graphene domain confined within a region bounded by neighboring larger graphene domains obtained as a function of time *t* during graphene growth. The domain appears fuzzy during early stages of growth. We estimate the rates of the domain motion to be between 10^–6^ and 10^–5^ m/s on both Cu and Au (see the SI for details). The domains appear sharper at later times, suggestive of reduced wobbling, which is directly correlated with the decreasing distance between the domains. During wobbling, the domains avoid merging and remain separate. As the domains grow larger, they inevitably attach rapidly either to the surrounding larger domain wall or coalesce with other adjacent domains (Figure 1b, Figure 2a). When attachment occurs, it disrupts the regular spacing of the domains. We suggest that the wobbling and the attachment of domains are consequences of repulsive and attractive forces, respectively, between the domains and their local environment.

**Figure 2:**
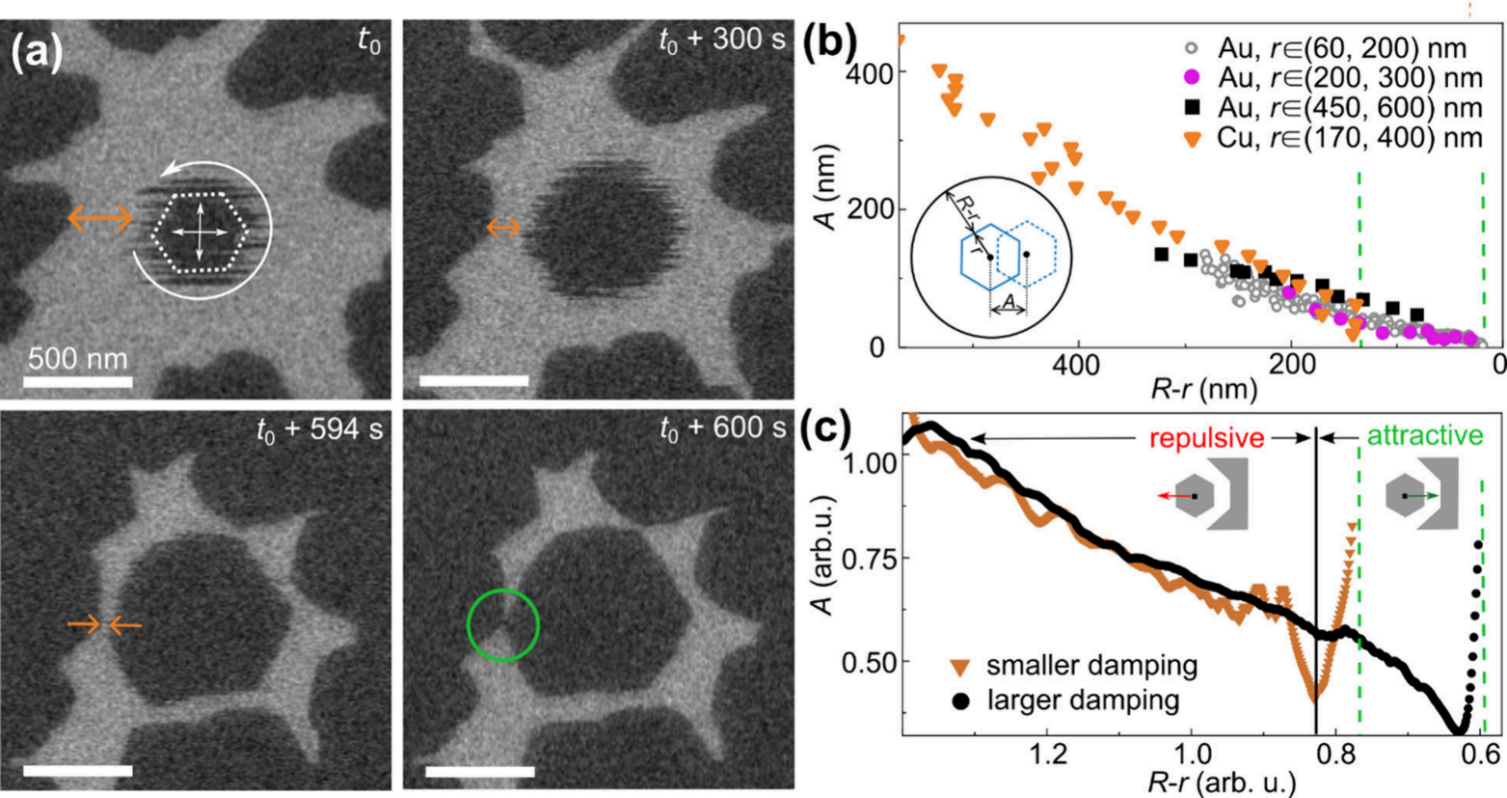
Graphene domain oscillations on molten metal. (a) Series of in situ SEM images (Movie S5 in the SI) obtained during graphene growth on a liquid gold using 15 Pa C_2_H_4_ at *T* = 1313 K. The dotted hexagon in the first image in panel a highlights a wobbling domain with fuzzy contours. The arrows within the hexagon and curve arrow indicate translational and rotational motion of the domain, respectively. A green circle marks a point of contact between the floating domain and the enclosing boundary. (b, c) Plots of experimental and simulated wobbling amplitudes *A* vs bare liquid spacing (*R*–*r*), indicated by the orange arrows in panel a. We define *r* (= $\sqrt{S/\pi }$ ) as the orientation-averaged size of the domain of area *S* and *R* as the distance between the domain’s center-of-mass to the surrounding boundary. The data plotted in panel b are obtained from graphene domains of different sizes growing on molten Au with 15 Pa C_2_H_4_ at *T* = 1313 K and on molten Cu with 1.2 × 10^–2^ Pa C_2_H_4_ at *T* = 1373 K. The *r* values in parentheses correspond to the initial and final sizes of the growing domains during the measurements. Additional details on the data extraction procedure can be found in the SI. In plot c, the black and orange curves are data extracted from simulations shown in Movies S6 and S7, respectively, carried out using larger and smaller damping coefficients. The solid black line marks the moment of the change from repulsive to attractive forces acting on the domain. The dashed green lines in panels b and c indicate the moment of attachment of the domains to other ones or to the enclosing boundary.

To better understand the dynamics of the domain motion, we have quantitatively determined the extent of wobbling from the in situ SEM image sequences. Figure 2b is a plot of the wobbling amplitudes *A* measured for growing domains of different sizes *r* and separation distances (*R*–*r*). We find that *A* decreases with decreasing free space (*R*–*r*). Importantly, all the data were obtained from domains of different sizes and shapes, in different environments, and on Cu and Au collapse onto a single curve (Figure 2b). Further detailed discussion of additional experimental observations is provided in the SI. A pronounced difference between the liquid Cu and Au substrates is that on Au, the wobbling ceases (*A*→0 in Figure 2b) at a critical distance *d*_min_ (*R*–*r*) of 50 ± 20 nm; for Cu, the critical distance is larger, approximately 140 ± 60 nm, suggesting that the balance between attractive and repulsive forces between the domains is reached sooner on Cu. As indicated by the dashed vertical lines in Figure 2b, domain coalescence follows, but the previously established assembly of the domains is always disrupted due to a renewed rapid motion of the domain just before coalescence (Movies S3 and S5).

Our in situ observations of graphene rheotaxy reveal self-assembly (Figure 1) of individual graphene domains into semiregular patterns. More interestingly, we find that the domains oscillate (Figure 2a) and reorient (see Figure 3a) during self-assembly. To explain these observations, we have set up an analytical model of surface undulations (manifested as surface waves with a wavenumber *k*) that act on floating 2D domains surrounded by stationary domains (see Modeling in the SI). The existence of surface undulations on liquid surfaces has been confirmed by X-ray diffraction.^32^ Undulations of the liquid surface can form either due to thermal fluctuations of the liquid surface^32–34^ or electrostatic interactions between graphene and the molten metal,^35^ generating long-range interactions over large-areas.^36,37^ The related deformation of the liquid surface exerts a mechanical force on the floating object, making it move and rotate, as has been demonstrated for colloidal microparticles.^36,38^ Given that the surface undulations induce surface roughness (1.5–3 Å) comparable to the gap between the floating graphene domain on liquid surface,^20,39–41^ we assume similar interaction between surface undulations and graphene.^40^ These surface undulations are referred to as “capillary waves” in the literature due to their similarity to capillary forces, although their origin is different. To avoid confusion, we will refer to the surface undulations as capillary waves in the following text. An inherent feature of capillary waves present in a confined space is a limited number of waves with certain *k*-vectors at each side of the floating graphene domain. Hence, the waves push the domain in certain directions (due to the absence of counteracting waves on the other side of the domain) and, potentially, induce a torque. The alignment of parallel long edges seen in our and others’ experiments^13,20^ is a characteristic consequence of capillary waves acting on floating objects. In the model, we assume that the waves appear only on a bare liquid, i.e. on the molten metal surfaces not covered by graphene. For simplicity, we also assume that the graphene is rigid. We note that the latter assumption is not realistic,^40^ but it does not affect the qualitative conclusions of the model. The waves are described by a solution of the 2D Helmholtz equation of the form, Δ*u*_*k*_ + *k* ^2^ *u*_*k*_ = 0, where *u* is the displacement with the same Dirichlet boundary conditions of the zero displacement for the boundary and for the edges of the graphene domains of interest. These solutions form a set of eigenfunctions belonging to specific eigenvalues of *k*. The wave amplitudes of every eigenfunction are given by a dispersion curve (similar to the theory of driven oscillations). Different waves can appear at different sides of the domains resulting in both repulsive and attractive interactions between domains. Since every wave carries certain momentum (and energy), the wave reflection is accompanied by a change in momentum manifested in forces *F⃗*_*i*_ ∝ $\underset{k}{\sum }{\left(\frac{\partial u_{k}}{\partial n_{i}}\right)}^{2}$ acting at *i*th point of the domain edge pushing perpendicularly against the domain edge. The translational and rotational motions of the domain are obtained by solving the equations of motion, *ma⃗* = – *bv⃗* + ∑_*i*_ *F⃗*_*i*_ and *Iε⃗* = – *βω⃗* + ∑_*i*_ *r⃗* × *F⃗*_*i*_, respectively, along the entire domain edge. In the above equations, *m*, *a⃗*, and *v⃗* are the mass, acceleration, and velocity of the graphene domain, respectively; *I*, *ε⃗*, and *ω⃗* are the moment of inertia, angular acceleration, and angular velocity, respectively, with respect to the center of mass of the domain. The parameters *b* and β represent the linear and angular damping constants, respectively, and are a measure of the dynamic viscosity of the molten metal.

**Figure 3:**
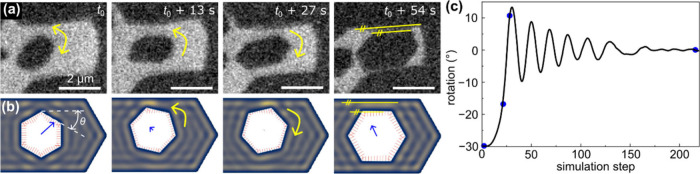
Reorientation dynamics of graphene domains on molten metal. (a) Typical in situ SEM images (full movie available as Movie S4) acquired from molten Cu at *T* = 1373 K during graphene growth using 1 × 10^–2^ Pa C_2_H_4_. Yellow arrows show directions of the domain rotations. Within *t*_0_ + 54 s, the domain attains a stable position, which is with the longest edges parallel to the boundary, as indicated by the yellow parallel lines. (b) Sequence of simulated images of a hexagonal domain floating on a liquid within nearly hexagonal compartment (see full Movie S6). Dark blue and yellow colors of the simulated liquid denote low and high amplitudes, respectively, of the waves emerging on the surface of the liquid. Lengths and directions of blue arrows represent magnitudes and orientations of the forces acting on the growing domain. Short red lines represent forces at individual positions around the domain edges. (c) Plot of domain orientation θ (defined in panel b) as a function of simulation time. Solid blue circles in the plot correspond to the images in panel b.

Our model qualitatively reproduces most of the experimentally observed behaviors. We have simulated the wobbling of floating domains within confined spaces, mimicking the experimental geometries. Figure 2c is a plot of calculated displacements of the domains as a function of the free (i.e., the region not covered by graphene) liquid surface, (*R*–*r*). The simulated plot is qualitatively similar to the experimental data in Figure 2b. As the domain grows larger in time, (*R*–*r*) decreases and the domain is dragged out of the near-equilibrium position due to rapidly increasing attractive forces between its edges and the nearby boundary. When attractive forces on one side of the domain prevail, the domain moves toward and eventually attaches to the adjacent boundary. Before coalescence, the domain assembly is disturbed due to additional torque forcing the moving domain to rotate during attachment (see Movie S6). This behavior is seen also in experiments (Figures 1b and 2a and Movies S3 and S5), even though the model predicts much more pronounced instability before coalescence than observed experimentally.

Movies S6 and S7 show the behavior of hexagonal domains on molten surfaces with different magnitudes of damping forces. The damping is a measure of viscosity, which is for liquid Au at 1373 K, ≈1.35× larger than that for liquid Cu at the same *T*.^42,43^ The black and orange curves in Figure 2c, respectively, correspond to larger and smaller damping values. Despite different damping, the scaling profiles of domain displacements vs (*R*–*r*) are nearly identical. More importantly, the domain attached sooner in case of smaller damping, as observed in experiments for the domains on liquid copper as compared to liquid gold (Figure 2b). This behavior suggests that the observed domain dynamics including the critical distance for attachment of the domains depend also on material parameters other than the viscosity of the molten metal. Further detailed modeling is necessary to elucidate the issue.

When a domain is confined in an enclosed space, formed for example by other domains, it rotates to attain a preferred position, which for hexagonal shapes is with their sides parallel to each other. This is experimentally demonstrated in Figure 3a, which shows rotation of an isolated hexagonal graphene domain floating on a liquid copper. The translational and rotational motion (see Movie S4 and also Movie S8) is restricted by the repulsive interactions arising from the presence of other domains in vicinity. This phenomenon is also captured in our modeling. Figure 3b shows a series of simulated images of a hexagonal domain floating and rotating on a liquid surface. The data are extracted from Movie S6 generated using our model. In agreement with the experimental observations, the domain rotates and reorients itself until it attains a metastable position, which is with its sides parallel to the domain boundary (Movie S4 and S8), ideal for seamless assembly. However, note that the model assumes perfectly smooth domain edges. A significant edge roughness, as sometimes observed in experiment, may prevent the parallel positioning of the domain edges.

Finally, we comment on the validity and significance of our model based on capillary waves. A direct observation of capillary waves on molten metals is beyond time and lateral resolutions of both SEM and AFM used; nevertheless, our data acquired with much higher lateral resolution than previous in situ optical microscopy-based data^20^ demonstrate that the distances between metastable floating domains are in nanometer range, which cannot be explained by electrostatic, van der Waals and capillary forces alone.^20,44,45^ We provide additional discussion of the other interactions in the SI. The observed reorientation of domains during growth can be attributed to the operation of capillary waves and electrostatic forces. Although the capillary waves arise from a stochastic process, the boundary conditions posed by the graphene domain edges selectively restrict certain wavelengths of the generated standing waves. Our model based solely on the capillary waves thus explains both the alignment (Figure 2) and rotation (Figure 3) observed in experiment despite the stochastic nature of the capillary waves; domain rotation occurs due to anisotropy in wavevectors that fit in between the domains that are not parallel. This effect is visible in our simulated movies, which show changes in amplitudes of capillary waves across the edges of misoriented domains. The model also nicely replicates more complex systems, e.g., coordinated behavior of many domains as observed experimentally in Movie S3 (see simulated Movie S9) and attractive interaction at the larger scale (see Figure S17 and Movie S10). Quantitative description of the observed phenomena requires detailed knowledge of the relation(s) between damping constants, viscosities and densities of the molten material, rigidity of the graphene, and dispersion curve of the surface waves,^46^ which is beyond the scope of our work. We note that the model does not include the influence of electric dipole interactions, which increase the repulsive interaction between domains and become more prominent with increasing domain size. Hence, we expect that the role of dipole–dipole interactions will be more important for graphene domains that are larger than those observed in this study^13,20^ and may be incorporated into our model to describe the domain behavior at different scales. We expect that the dipole–dipole interactions may be significant at the coalescence stage, contributing to the stabilization of domains before attachment. Therefore, accounting for electrostatic forces could potentially explain the difference between the model simulations (Figure 2c) and our experiments (Figure 2b) in the coalescence stage.

In conclusion, we have investigated the phenomenon of domain dynamics occurring during the chemical vapor deposition of graphene on molten metals such as copper and gold. Our in situ SEM observations reveal that individual graphene domains oscillate and rotate during growth as a means to arrange themselves in spatially periodic arrays. Based on the time-resolved measurements of the domain dynamics as a function of their sizes and HT-AFM data of the graphene/molten-metal topography, we propose a continuum model that relies on the presence of capillary waves. Our model is material independent, and hence is applicable to predict and explain self-assembly of 2D layers and even 3D crystals on surfaces of any liquids and weakly interacting materials capable of producing surface undulations. Our experimental and modeling data reveal that the choice of appropriate liquid substrate material facilitates stable oscillations of domains over longer periods of time and hence is critical for achieving self-assembly and seamless stitching of domains, essential for large-area rheotaxy of single-crystalline sheets of 2D layers.

## Supplementary Material

























## Data Availability

The data underlying this study are openly available at https://zenodo.org/records/14883846.

## Notes

Materials and Methods, model description, further considerations accounting for other interactions, discussion of possible presence of meniscus around graphene domains floating on a liquid metal, supplemental experiments for discussion of wobbling, discussion of attractive interaction between the domains mediated by capillary waves, Tables S1–S4, Figures S1–S16, and movie descriptions (PDF)
Movie S1 showing experimental workflow (AVI)
Movie S2 showing growth of graphene domains on solid gold (AVI)
Movie S3 showing alignment of graphene domains on liquid gold (AVI)
Movie S4 showing rotating graphene domain in an enclosed space (AVI)
Movie S5 showing graphene domain enclosed inside a compartment (AVI)
Movie S6 showing simulation of a domain behavior on a liquid (MP4)
Movie S7 showing simulation of a domain behavior on a liquid with different viscosity (MP4)
Movie S8 showing domain assembly inside a graphene compartment on liquid Cu (AVI)
Movie S9 showing assembly of multiple interacting domains (MP4)
Movie S10 showing attractive force between the floating domains (MP4)
Transparent Peer Review report available (PDF)
