# Supplementary material for: Capillary Wave Driven Dynamics of Graphene Domains during Growth on Molten Metals
Source: J Phys Chem Lett. 2025 Sep 17;16(38):10020–6. doi: 10.1021/acs.jpclett.5c02321 (PMC12478858; doi:10.1021/acs.jpclett.5c02321)
Supplement: Supplementary file 1 [file jz5c02321_si_001.pdf]

Supporting Information for

Capillary wave driven dynamics of graphene domains during growth on molten metals

Kristýna Bukvišová[1,2], Radek Kalousek[1,3], Marek Patočka[3], Jakub Zlámal[1,3], Jakub Planer[1], Vojtěch Mahel[2,3], Daniel Citterberg[3], Libor Novák[2], Tomáš Šikola[1,3], Suneel Kodambaka[4], Miroslav Kolíbal[1,3*]

[1]CEITEC BUT, Brno University of Technology, Purkyňova 123, 612 00 Brno, Czech Republic

[2]Thermo Fisher Scientific, Vlastimila Pecha 12, 627 00 Brno, Czech Republic

[3]Institute of Physical Engineering, Brno University of Technology, Technická 2, 616 69 Brno, Czech Republic

[4]Department of Materials Science and Engineering, Virginia Polytechnic Institute and State University, Blacksburg, Virginia 24061, United States

Corresponding author: kolibal.m@fme.vutbr.cz

Table of Contents

Materials and Methods....................2

Model description 7

Further considerations accounting for other interactions....................9

Discussion of the possible presence of meniscus around graphene domains floating on a liquid metal 18

Supplemental experiments for discussion of wobbling 21

Discussion of attractive interaction in between the domains mediated by the capillary waves.....27

Description of Supplementary Movies 28

Additional references 30

Materials and methods

Observation of graphene growth in a MicroReactor. All the graphene growth experiments on solid and molten gold are carried out using a MicroReactor (see Fig. S1A) in a Thermo Scientific Helios 5 UC system equipped with a scanning electron microscope (SEM) and focused ion beam (FIB) milling. Gold samples ($40 \times 40 \times 30$ μm^3) were extracted from a 50 μm-diameter gold wire (Agar Scientific, 4N purity) *via* FIB milling in the Thermo Scientific Helios 5 Plasma FIB. In order to avoid surface contamination, instead of typical ion-beam-induced deposition, we have utilized sputtered gold atoms that redeposit onto the nanomanipulator during ion beam bombardment of the Au sample, a procedure commonly denoted as attachment by redeposition. The gold samples were then positioned on the NanoEx Micro Electro Mechanical System (MEMS) heating chip and glued by ion beam-induced deposition. The chips were then placed in the MicroReactor and loaded into Helios 5 UC DualBeam equipped with a custom-built gas feeding system with individual inlets for dosing C_2H_4, O_2, and H_2 gases. Au pieces were first annealed in oxygen at temperatures T between 1073 and 1273 K for a variable amount of time (units of hours) to remove carbon deposits and other surface contaminants. Then, the Au was melted in ~10 Pa H_2 environment by heating above 1337 K. Any contaminants that emerged on the surface of the molten Au were then removed by gentle Ga ion beam milling in the FIB at room temperature. Finally, the samples were annealed sequentially in oxygen and hydrogen atmospheres until visually clean molten Au droplets are obtained. Graphene growth experiments are carried out by dosing ethylene (C_2H_4) gas at the desired temperature.

SEM imaging conditions. SEM images are acquired in the secondary electron mode using 1-20 kV accelerating voltages and electron beam currents between 50 pA and up to 1 nA to minimize charging and thermal gradients possibly caused by the electron-beam. To test for the effects of electron-beam on the observed phenomena, graphene growth rates and graphene domain motion were compared during continuous and intermittent imaging. We did not observe any electron-beam-induced effects on the growth kinetics. The domain wobbling occurs independent of the beam parameters and while we cannot prove their existence with the beam off, we find that the domains remain in place without attachment to their surroundings irrespective of the scanning speed and with the beam on or off (see Fig. S13). Additional experiments (Fig. S15) demonstrate that the trajectories of the domains deduced from image sequences taken under different beam conditions remain similar.

Observation of graphene growth in UHV SEM. A modified Tescan ultrahigh vacuum (UHV, base pressure < 10^{-6} Pa) SEM was used for all the experiments of graphene growth on solid and liquid Cu. Copper wire (EM-Tec, 3N purity) was wrapped around a V-shaped flattened platinum wire (Goodfellow, 4N purity) that was resistively heated by passing current in the UHV SEM. The temperature was monitored using a pyrometer (Micro-Epsilon thermometer CTLaser M3), calibrated by assigning Cu melting point to the temperature at which the melting was observed in the SEM. Melting was performed in 5×10^{-3} Pa hydrogen atmosphere to mitigate oxidation of copper. Graphene is grown by slowly introducing ethylene after closing the hydrogen valve. Because of the low precursor pressures during the reaction, nucleation took place tens of minutes after the first introduction of ethylene, when the pressure was fairly stabilized. The molten copper evaporated rapidly, so the time available for observation of the liquid surface was limited. Furthermore, increasing the temperature above the melting point induced mixing of platinum

substrate with the copper droplet. Imaging was performed at 5 kV with a beam current in the range 300 pA to 1 nA, with no observable effect of the electron beam.

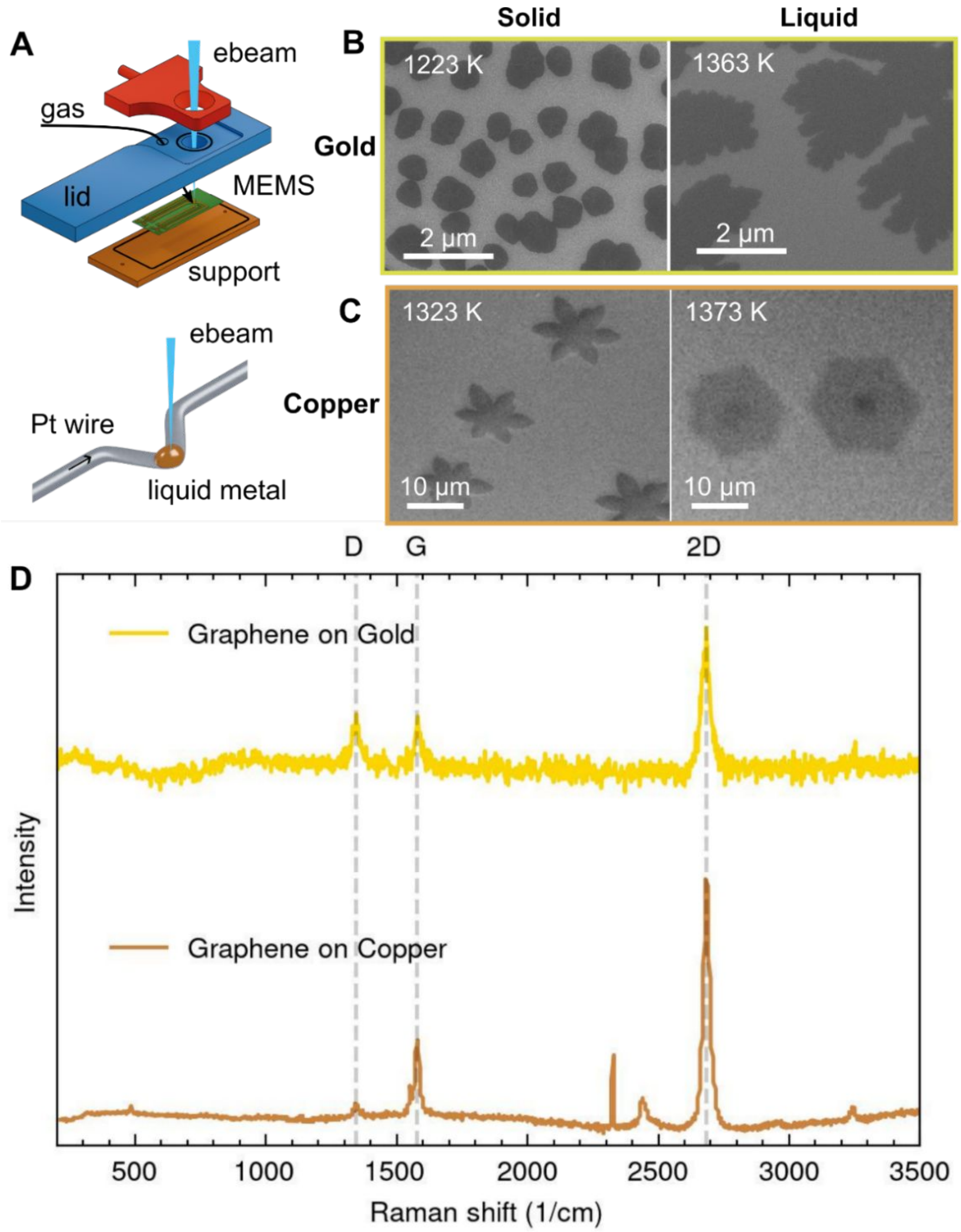

Figure S1. Domain shapes and Raman spectra of graphene. (**A**) Schematics of the MicroReactor design (top) and resistively heated Pt wire support for the growth of graphene (bottom), respectively, on Au at high fluxes and on liquid Cu droplets at low fluxes in UHV SEM. (**B,C**) *In situ* SEM images of graphene domains acquired from Au (**B**) and Cu (**C**) during exposure to ethylene (C_2H_4) at 30 and 1.2 $\times 10^{-2}$ Pa for gold and copper respectively on solid and liquid surfaces at temperatures T indicated in the panel. Electron beam imaging conditions: 10 keV, 0.2 nA (**B**) and 5 keV, 1 nA (**C**). (**D**) Raman spectra of graphene grown on Au and Cu, obtained after rapid (50 K/s) cooling of the as-deposited samples to room-temperature. Higher intensity D peak in the Raman spectra from graphene on Au is likely a result of large number of structural defects and due to smaller grain size of graphene on liquid Au.

Image processing. Growth of graphene domains and areal coverages are tracked using a Python script. First, the image signal is improved, by removing noise, using Block matching and 3D filtering (bm3d package). Background was removed using the Rolling ball background subtraction (part of scikit-image package). Finally, the image was thresholded using the Otsu's method and the segmentation was visually checked. For the size evolution of a specific domain, the segmentation was manually processed using the ImageJ software.

Analysis of the domain oscillation dynamics was carried out by a combination of automated script and manual processing. For each frame, we calculated the extrapolated radius of the domain based on the evolution of graphene growth rate surrounding the domain and its size after attachment. Further, we determined the envelope of the oscillatory motion, from which we derive its amplitude, and the size of the uncovered surface surrounding the domain. More details can be found below in the section titled: SEM image analysis: Data extraction procedure.

In situ high-temperature atomic force microscopy (HT-AFM). It is important to note that the *in situ* HT-AFM measurements are critical to assess the surface meniscus of the molten metal as *ex situ* characterization of graphene-metal interfaces at room-temperature can lead to misleading deductions (see Fig. S8).

High-temperature topography measurements by AFM were performed using LiteScope AFM-in-SEM microscope with Akiyama self-sensing probe (resonant frequency ~45 kHz, spring constant ~5 N/m, tip radius <15 nm) in the frequency-modulated tapping regime in the high vacuum of Versa FIB-SEM. The sample (FIB-prepared Au particle) was melted by the MEMS chip, graphene crystals were grown on top of it by CVD by introducing 65 Pa of ethylene into the SEM chamber for 5 minutes. After that, the ethylene was pumped, and the sample was kept under high vacuum in the molten state for the AFM measurement. Since the AFM tip, a solid, is likely at a lower temperature than the molten metal surface, prolonged tip-surface contact may lead to local solidification. Therefore, special precautions were taken to avoid this possibility. During the measurement, the probe was approached to the sample using a comparatively low setpoint of 2 Hz (commonly used values range from 5 to 20 Hz or even higher) in order to decrease the tip-sample force as much as possible. In some instances, the setpoint (i.e., the tip-sample force) was adjusted during scanning to reveal how the observed height of the step between liquid gold and graphene depends on the tip-sample force. Nevertheless, it must be noted that the apparent graphene height above the liquid may be misleading. In Ref. 1 the phenomenon is explained thoroughly, and it is claimed that unless special care is taken, the step heights obtained can vary significantly among images. In line with this study, we have observed that changing the setpoint of our measurement gives different graphene heights. Therefore, we do not discuss the graphene height above the liquid, but the height modifications of the liquid.

Raman spectroscopy. Raman analysis was performed with Witec Alpha 300R using 0.2 mW excitation laser at wavelength of 532 nm in backscattering configuration.

Density functional theory (DFT) calculation procedure. All DFT calculations were performed with the Vienna ab initio simulation package (VASP)[2] using the projector-augmented wave method (PAW)[3] for treating core electrons. Eleven valence electrons for gold and copper, four valence electrons for carbon, and one valence electron for hydrogen are described with a plane-wave basis set with an energy cut-off set to 450 eV. The Perdew-Burke-Ernzerhof functional was adopted for the exchange-correlation energy, supplemented by the Grimme pairwise dispersion correction D3.[4]

The structural and electronic calculations were performed in two steps: The gamma-point calculations were employed for initial optimization, and the process was terminated when the residual forces acting on ions decreased to values below 0.04 eVÅ^{-1}. Subsequent electronic structure calculations involved a Gamma-centered 2×2×1 Monkhorst-Pack grid[5] for Brillouin zone sampling, and the number of FFT grid points was increased by 30%.

For isolated graphene domains adsorbed on the gold and copper surfaces, the substrates were represented using 4-layered (111) slabs within a rectangular supercell with the matrix notation (11 0 | -6 12). The dimensions of the supercells were approximately 26.2×27.7 Å^2 and 30.1×31.9 Å^2 for the copper and gold surfaces, respectively. The structures for the full graphene monolayer were adopted from ref. 6. The periodically repeated sheets were separated by 15 Å thick vacuum layer and all calculations included dipole corrections to both the potential and energy. Bader charge analysis[7] was conducted to assess the induced dipole charge between a metal substrate and a graphene domain.

SEM image analysis: Data extraction procedure

The image sequencies were processed according to the description below, and the following quantities were extracted (as a specific example, Fig. S2 shows the analysis if image sequence in Fig. 2).

On liquid gold:

- **Domain radius** *r* was extrapolated back in time based on the growth rates of the domains that were static (e.g. pinned to a stationary edge). As soon as the domain attached to the surrounding domains, we measured its size and used it for the retrospective analysis, based on the evolution of growth of the static graphene surrounding the oscillating domain. We checked that the above-mentioned approach gives nearly identical results as when the graphene area is deduced from image contrast in every frame, deviating only for the initial stages, when the domain is very small and oscillates vigorously.
- **Amplitude of motion**, *A*: For each frame, we measure the envelope of the smeared domain. We used two strategies, manual segmentation and automated image processing in a Python script. In the script, we used a Gaussian kernel 1x3 pixels that induced “smearing” in vertical direction and then the domain envelope was thresholded. There was a good agreement between the data extracted by both approaches, with the exception of initial stages of growth. There, the domain size was small and the smeared domain appeared discontinuous in the image and could not be analyzed by the script. Therefore, the data shown is the one acquired manually.
- **Radius** *R* of the area in which the oscillating domain is enclosed: For each image in the sequence, we inscribed a largest circle in which the domain could oscillate without coming into direct contact with the surrounding domains. *R* is the radius of the inscribed circle.
- **Minimum distance,** defined as R-(r+A).

On liquid copper:

- Thanks to the possibility to capture arbitrary scan windows, the frame-time was reduced and we did not analyze smeared domains like in the case of liquid-gold-related datasets. Instead, we are able to directly measure domain parameters in each frame (*r*, *R*). When large-area scan was performed, the domain was smeared in an identical way as in the MicroReactor datasets.

- However, the time between two subsequent images is larger compared to those observed on molten Au, so we have relatively fewer data on the domain movement. The amplitude A was determined as the envelope of center-of-mass evolution in time.

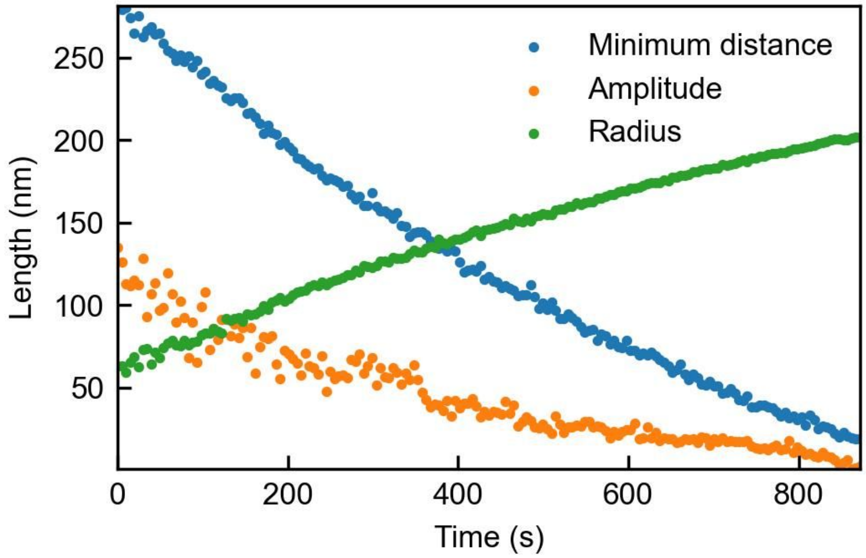

Figure S2. Quantification of the image sequencies of oscillating domains. Here, the results of processing of image sequence shown in Fig. 2 (Movie S5) are shown (graphene growth on a liquid gold using 15 Pa C_2H_4 at T = 1313 K). Several quantities can be drawn from the image sequences, as described above in the text. Analysis of the oscillating domains varied between the Cu and Au datasets because of varying frame rates, inherent to different microscopes that were used to capture the growth (see above).

Domain displacement rates. The rates of domain motion are between 10^{-6} and 10^{-5} m/s, as deduced from the scanning rate of the electron beam, for both copper and gold surfaces, despite different beam scanning strategies. The rates are estimated to be in this interval by the following procedure. For the lowest dwell times possible (50-100 ns) and low magnifications, the 'free' domains appear sharp, meaning that the time it takes to scan over the particle is comparable or smaller than that required for the domain to move away from the scanned area. This gives an estimation for the upper boundary for the velocity interval. At longer dwell times, we observe displacement of the domain in between two consecutive scans. This displacement, together with the frame time, determines the lowest possible velocity of the domain.

Model description

<u>Capillary waves.</u> We concentrate only on mechanical waves that appear on a free liquid surface mostly due to thermal fluctuations. We assume that these mechanical waves are the only cause of forces acting on the domains. Different waves can appear at different sides of the domain manifesting in both repulsive and attractive interaction between domains (see Fig. S3). This interaction has an analogy in Casimir effect well-known in quantum field theory.

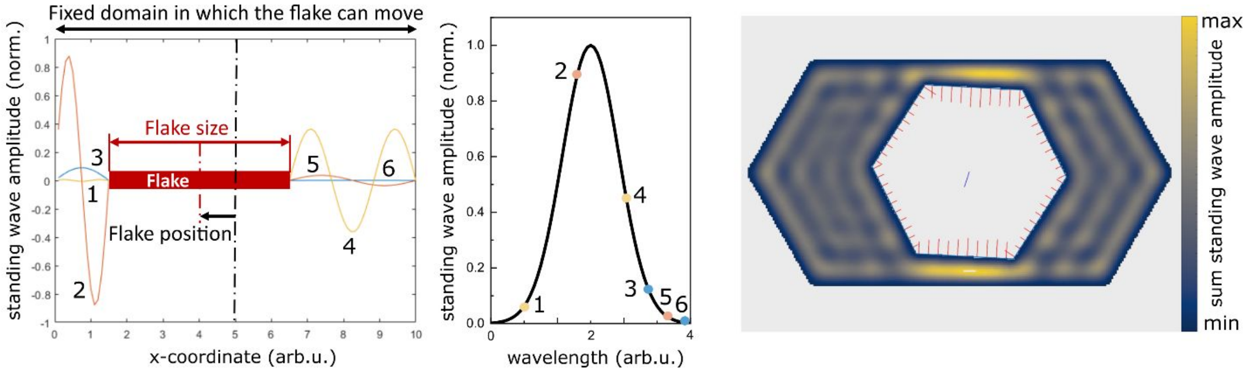

Figure S3: **Explanation of the model of capillary waves**. Left: A 1D sketch illustrating the domain floating on a liquid with undulated surface. The wave dispersion curve is shown in the middle, with 6 specific wavelengths referring to the waves depicted in the sketch on the left (marked by numbers). Right: The simulations presented in the main text are done in 2D, where we plot the sum intensity of all the surface waves. In this case, due to a confined space in between the compartment edges and the top and bottom edges of the domain, the biggest forces are acting there (red lines), resulting from the waves of highest amplitudes (bright yellow regions).

Let us consider the free liquid surface as a region where the mechanical waves can appear while the surface covered by the domain is completely solid (e.g., we treat the simplest case where elasticity of the graphene domains is neglected). The waves are described by a solution of the 2D Helmholtz equation (stationary wave equation) where the domain edges represent a Dirichlet boundary condition with zero displacement. The amplitudes of the waves with a specific wavelength (wavenumber) are given by a dispersion curve (similar to the theory of driven oscillations), see Fig. S3. The boundary condition is a result of the wave reflection from the domain edge. Since every wave carries certain momentum (and of course energy), the wave reflection is accompanied by the change of momentum manifested in forces pushing perpendicularly against the domain edge. These forces have always the same direction regardless of the instant direction of the wave displacement near the edge, therefore, let us consider that the force is proportional to the square of the directional derivative of a wave at the edge point with respect to the direction perpendicular to the edge. The domain motion (both translational a rotational) is obtained by solving an equation of motion containing all forces along the entire edge.

A solid region where the domain(s) is/are considered to move is selected. This region represents the surrounding domains that are already immobile. The edges of this region are fixed and are defined by the same Dirichlet boundary condition (zero displacement) as well as the domain edge. The domain of a certain shape, size, and orientation is placed in the region. In the first step, the 2D Helmholtz equation

$$\Delta u_k + k^2 u_k = 0$$

for all wavenumbers k is solved inside the region with the Dirichlet boundary conditions of zero displacement mentioned above. As expected, these solutions form a set of eigenfunctions belonging to specific eigenvalues of k. The amplitudes A_k of every eigenfunction are chosen according to a unique and temporarily constant dispersion curve defined by a specific position and width of its peak. The magnitude of the force $\vec{F}_i$ acting perpendicularly against the i-th point of the domain edge is proportional to the sum

$$\sum_k \left(A_k \partial_{\vec{n}_i} u_k\right)^2$$

where $\partial_{\vec{n}_i}$ is the directional derivative with respect to the direction $\vec{n}_i$ perpendicular to the edge. The translational equation of motion is given as follows:

$$m\vec{a} = -b\vec{v} + \sum_i \vec{F}_i ,$$

where m is the domain mass, $\vec{a}$ and $\vec{v}$ are the domain acceleration and velocity, respectively, and b is the drag constant. The rotational equation of motion reads

$$I\vec{\varepsilon} = -\beta\vec{\omega} + \sum_i \vec{r_i} \times \overrightarrow{F_i},$$

where I is the moment of inertia, $\vec{\varepsilon}$ and $\vec{\omega}$ are the respective domain angular acceleration and angular velocity, all with respect to the domain center of mass, β is the angular drag constant, and $\vec{r_i}$ is the position of i-th point of the domain edge with respect to the domain center of mass. In the next step, these two equations of motion predict small changes of the position and orientation of the domain resulting in slightly different set of eigenvalues k and eigenfunctions u_k when solving the 2D Helmholtz equation for the new scenario. The numerical simulation of the domain motion consists in repeating these two steps in which a gradual growth of the domain can be taken into account as well.

Further, we describe the meaning of used quantities and functions. Displacements u refer to surface waves that origin in thermal fluctuations (where amplitudes of several Ångstroms have been reported in experimental works).[8,9] Additionally, the displacements may be related to the effects of adsorption/desorption kinetics within a smooth transition region between the liquid and vapor phase, which can have such short wavelengths (~ 100 nm). The damping constants b and β are principally related to the dynamic viscosity of the liquid metal, resulting in losses in the mechanical energy of the domain. The dispersion curve[10] results from the following consideration: the liquid behaves as a resonant system exhibiting a certain resonant frequency. This system is driven by intrinsic thermal fluctuations at various frequencies causing high amplitudes of oscillations at frequencies close to the resonant one, while very low amplitudes at different ones.

Further considerations accounting for other interactions.

Apart from Casimir-like surface undulations, we have considered electrostatic dipole interaction (repulsive), van der Waals forces (attractive), and capillary forces. In this section, we discuss in detail a possible contribution of these interactions. We first estimate the dipole charge induced at the interface between the floating graphene domain and a substrate by DFT and then demonstrate an analytical calculation of electrostatic potential energy of the charged domains. Then we compare this energy to $k_B T$ and show that the electrostatic forces alone cannot account for the observed behavior. Then, we combine the repulsive electrostatic interaction with an attractive van der Waals and demonstrate that the experimental results cannot be interpreted by these interactions neither.
We finish our discussion by an in-depth analysis of possible meniscus appearance in the experiments, which would be a fingerprint of macroscopic capillary forces. The capillary forces have been speculated to induce a nanoscale 'Cheerios effect'[11,12] for floating objects. But surface morphologies of graphene domains on molten metals measured using *in situ* HT-AFM and SEM (see figs. S1, S9) do not show menisci around the graphene domains, indicating that classical capillary forces, if present, are negligible.

Estimation of the charge transferred to the graphene. We have used density functional theory (DFT) to quantitatively assess the charge density redistribution between a graphene domain and the metal substrate. To elucidate whether the graphene domains are H-terminated (Fig. S4A-C) or metal-terminated (Fig. S4D-F), we compare the calculated binding energy of the hydrogen atoms with the hydrogen chemical potential at experimental conditions (μ_{H2} = -2.08 eV for 1070 °C and 10^{-5}mbar H_2). The binding energy of the hydrogen atoms terminating a graphene domain is calculated to be -1.89 eV, i.e., close to the hydrogen chemical potential. Therefore, we conclude that DFT calculations do not exclusively prefer any of the terminations considered. The binding energy of hydrogen in the graphene domain on the copper substrate is reduced to -1.57 eV, showing that DFT prefers the Cu-terminated graphene domains. Since the induced dipole charge of the metal-terminated domains are calculated to be lower than the domains terminated with hydrogen, as discussed later, we will build a phenomenological model for the estimation of the induced dipole charge between the Au(111) and Cu(111) substrates and a hydrogenated domain. Here we assume that graphene domains interact via electrostatic dipole-dipole interaction.[13,14] Two effects contribute to the size of the induced dipole moment at the interface between a graphene domain and a substrate. The first, (fractional) charge transfer, is caused by different work functions of the metal and the graphene,[13,14] while the second one, known as the push-back effect, is induced by the electrostatic repulsion, where the graphene layer pushes a metallic charge density spillover back to the substrate.

To estimate the charge density redistribution upon adsorption in the graphene layer, we employ the modified Bader charge analysis: The Bader volumes are evaluated from the all-electron charge density of the system on a denser FFT grid. Then, we integrate the charge density difference $\Delta\rho$ (x,y,z) between the interacting and separated systems over the Bader volumes. Specifically, $\Delta\rho$ (x,y,z) is calculated as

$$\Delta\rho(x,y,z) = \rho_{substrate+flake} - \rho_{substrate} - \rho_{flake}.$$

To assess the validity of this approach, we compare the resulting induced dipole charge transferred from a substrate to a domain with the maximum of the cumulative charge transfer function $Q(z)$,

which contains contributions from both the fractional charge transfer and from the push-back effect. This is defined as

$$Q(z) = \iint_A \int_{-\infty}^{z} \Delta\rho(x,y,z) dA dz \,.$$

Calculated charge density differences and cumulative charge transfer functions for the graphene domains on the Au(111) substrate shown in Fig. S5 reveal the electron accumulation close to the substrate layer and electron depletion at the graphene domain. Qualitatively same behavior is also observed for the Cu(111) substrate.

Table S1 summarizes the dipole charge induced between the substrate and hydrogen-terminated graphene domains. As both approaches produce comparable results, with relative differences below 2.4% for Au(111) and 11.2% for Cu(111), we conclude that the modified Bader charge analysis is a reliable method for evaluating the distribution of the induced charge on the domain.

Next, we define a surface charge density $\sigma(R)$ of the redistributed charge inside the domain upon adsorption on a substrate using the calculated Bader charges. Independent of the size, the graphene domain can be divided into inner and outer regions where the induced dipole charge scales linearly with the number of atoms therein, only with different slopes, as shown in Fig. S6B,C for the Au(111) and Cu(111) substrates, respectively. Therefore, we assume that the inner region has the surface charge density σ_i and the outer region σ_o. Also, the width of the outer region is fixed to d for all domains independent of their sizes. Thus, $\sigma(R)$ can be defined for all calculated domains as:

$$\sigma(R) = \sigma_i \cdot \theta[(R_T - d) - R] + \sigma_o \cdot \theta\big[R - (R_T - d)\big],$$

where R_T is the radius of the domain and $\theta(x)$ denotes the Heaviside function. Integration of $\sigma(R)$ over the area, shown as dashed lines in Figs. S6E,F, yields the total charge induced in the pink circle, as depicted in Fig. S6A. This can also be expressed as a function of the number of atoms inside the circle, illustrated by the dashed lines in Figs. S6B,C under the assumption that the surface density of carbon atoms agrees with graphene, i.e. ~2.6 $Å^2$ per carbon atom.
Overall, three parameters were fit to reproduce the induced dipole charge obtained from the modified Bader analysis (points in Figs. S6B,C,E,F): the surface charge density of the induced dipole charge in the inner and outer regions of the domain σ_i, σ_o, and the width of the outer region d. The optimal parameters for all the domains and for the graphene sheet adsorbed on both Au(111) and Cu(111) substrates are summarized in table S2. There is only one set of parameters needed for all calculated domains on the Au(111) substrate, with σ_i deviating by 3% from graphene. The domains adsorbed on the Cu(111) substrate converge slower with σ_i to the value related to the graphene layer. Therefore, two sets of parameters are necessary: one for the smaller 3×3 and 4×4 domains and one for the 5×5 domain, which differs in σ_i by 3% relative to graphene. Graphene domains adsorbed on the Cu(111) substrate generally show a larger induced dipole charge compared to the Au(111) substrate. This can be understood from the band structure of the graphene sheet adsorbed on both substrates projected on the carbon p_z orbitals, as shown in Figure S6D. Our calculations show that the graphene sheet adsorbed on the Au(111) substrate shows a slight p-doping (i.e. increasing the induced dipole charge), while the Cu(111) substrate induces n-doping

in the adsorbed graphene sheet (decreasing the induced dipole charge), which qualitatively agrees with the available experimental data.[15,16] Since the contribution from the push-back effect always decreases the induced dipole charge, the resulting value is expected to be more negative for the Cu(111) substrate than for the Au(111) substrate.

Finally, we evaluate the position of the mirror charge by fitting the resulting dipole moment to the value obtained from the cumulative charge transfer function. The optimal separation distance is calculated to be 1.85 Å for Au(111) and 1.72 Å for Cu(111), with relative errors below 6%. These values are in agreement with the calculated plane-averaged charge density differences shown in Fig. S5. Furthermore, the dipole moment calculated from the cumulative charge transfer function is in perfect agreement with the compensating dipole moment of the self-consistent field SCF run, shown in table S3. Therefore, we conclude that there are no intrinsic dipoles present either in the slab or in the graphene domain.

This model is only valid for the hydrogen-terminated domains, which do not contain intrinsic dipole moments. However, the metal-terminated domains do not fulfil this assumption because of the significant bending observed in all structures (Figs. S4D-F). Instead of building a more complex model for the metal-terminated domains, we show in table S4 that the resulting dipole moments from the SCF runs are always smaller than for the hydrogen-terminated variations. This behavior can be explained by an increased charge transfer from the substrate to the layer arising from the compensation of the missing hydrogen bonds, which tends to counterbalance the push-back effect. Therefore, the metal-terminated domains are expected to interact weaker than hydrogen-terminated domains.

In summary, the induced charge for the hydrogen-terminated graphene domains can be described by a constant surface charge density σ_i over the whole area of the domain except for the 2.25 Å thick edge region with the charge density 1.7 - 2.2× higher. The values of σ_i presented in table S2 are for the largest calculated graphene domains in a good agreement (relative errors below 3%) with the calculated surface charge density for the graphene layer. The dipole charge induced in the graphene domain is accompanied by a mirror charge, separated by 1.85 Å and 1.72 Å on Au(111) and Cu(111) substrates, respectively. These values were chosen such that they reproduce the total dipole moment in the system obtained from the SCF run.

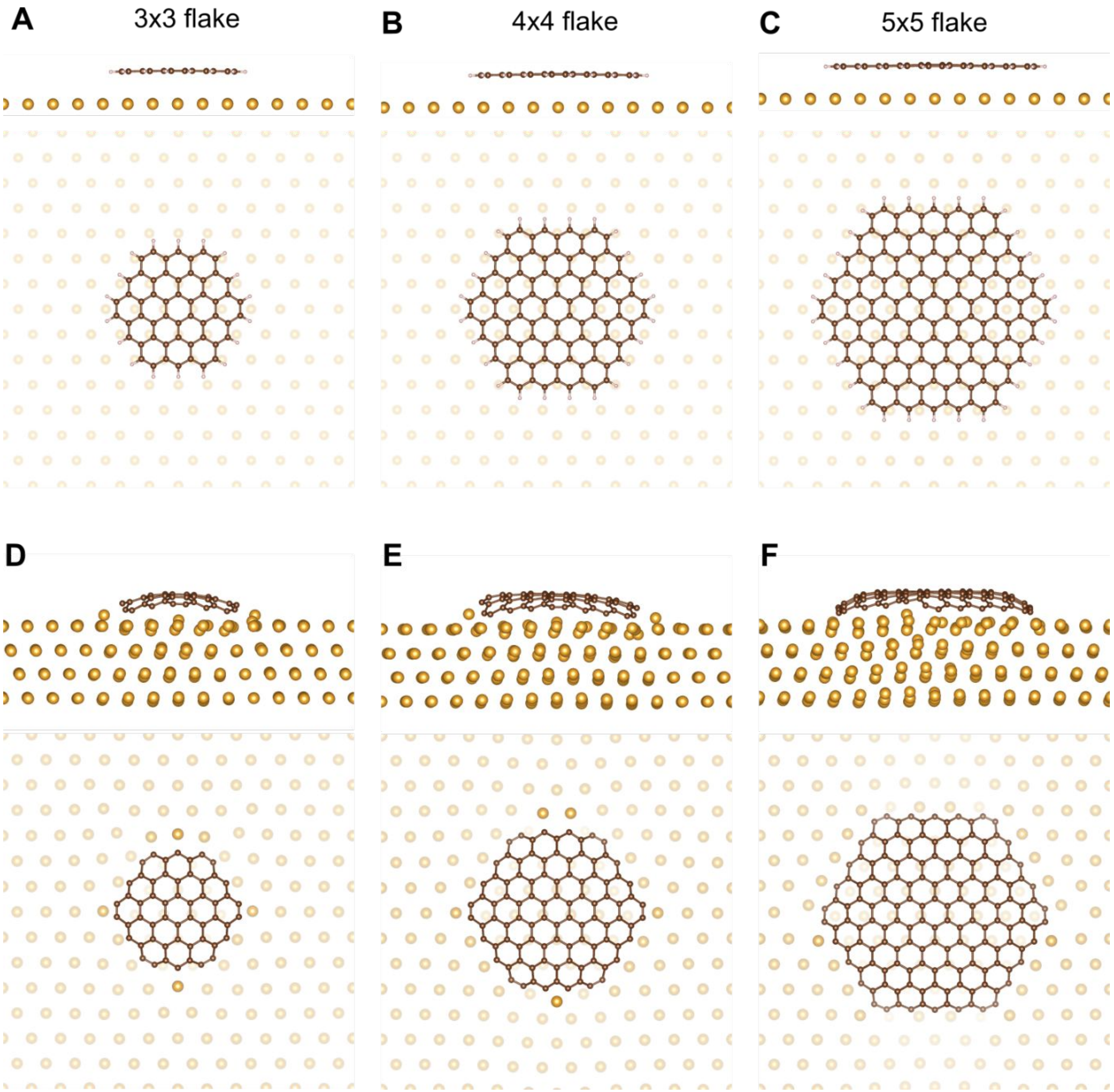

Figure S4: **Top and side views on the relaxed geometries of the hydrogen-terminated (A-C) and Au-terminated (D-F) graphene domains**. The calculated structures are hexagonally shaped with the side composed of 3 aromatic rings marked as 3×3 (**A, D**) up to 5 aromatic rings marked as 5×5 (**C, F**). Au-terminated domains are bent toward the substrate, causing significant buckling in the first layer of gold. Qualitatively similar structures were also obtained for the Cu(111) substrate.

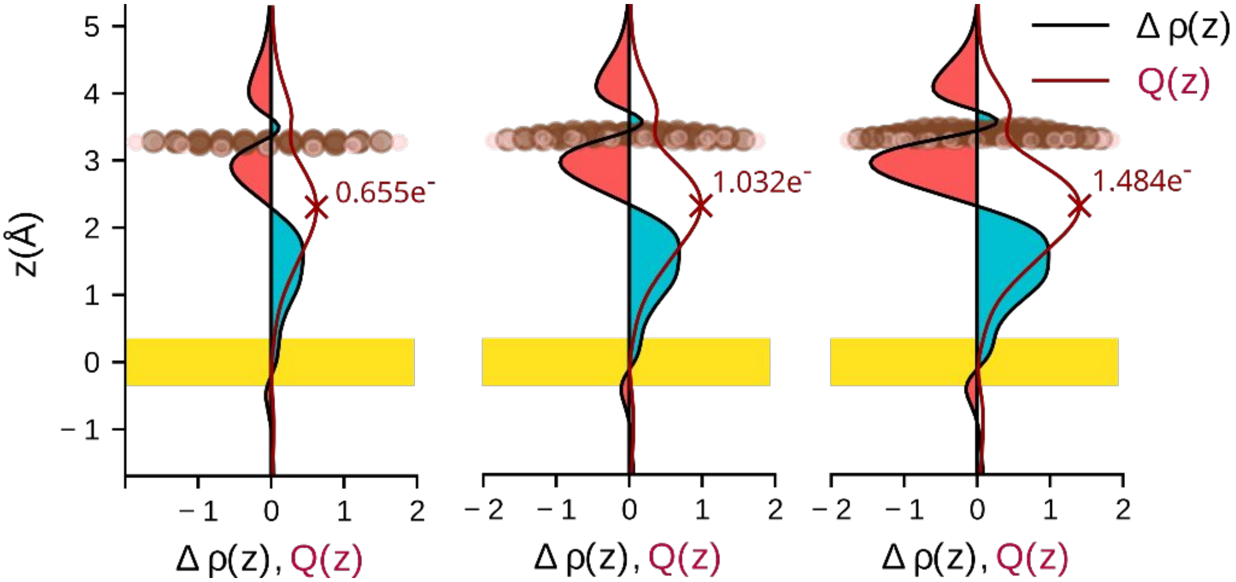

Figure S5: Plane-averaged charge density differences $\Delta\rho(z)$ and cumulative charge transfer functions $Q(z)$ for the graphene domains interacting with the Au(111) substrate with the size of 3×3, 4×4 and 5×5. Areas of electron accumulation are colored in blue and electron depletion in red. The position of the first substrate layer represented by the yellow rectangles is set to 0. The maximum of $Q(z)$ defines the number of electrons transferred from the domain to the substrate. Brown and white semi-transparent spheres at $z = 3.2$ Å denote z-positions of carbon and hydrogen atoms of the graphene domain, respectively.

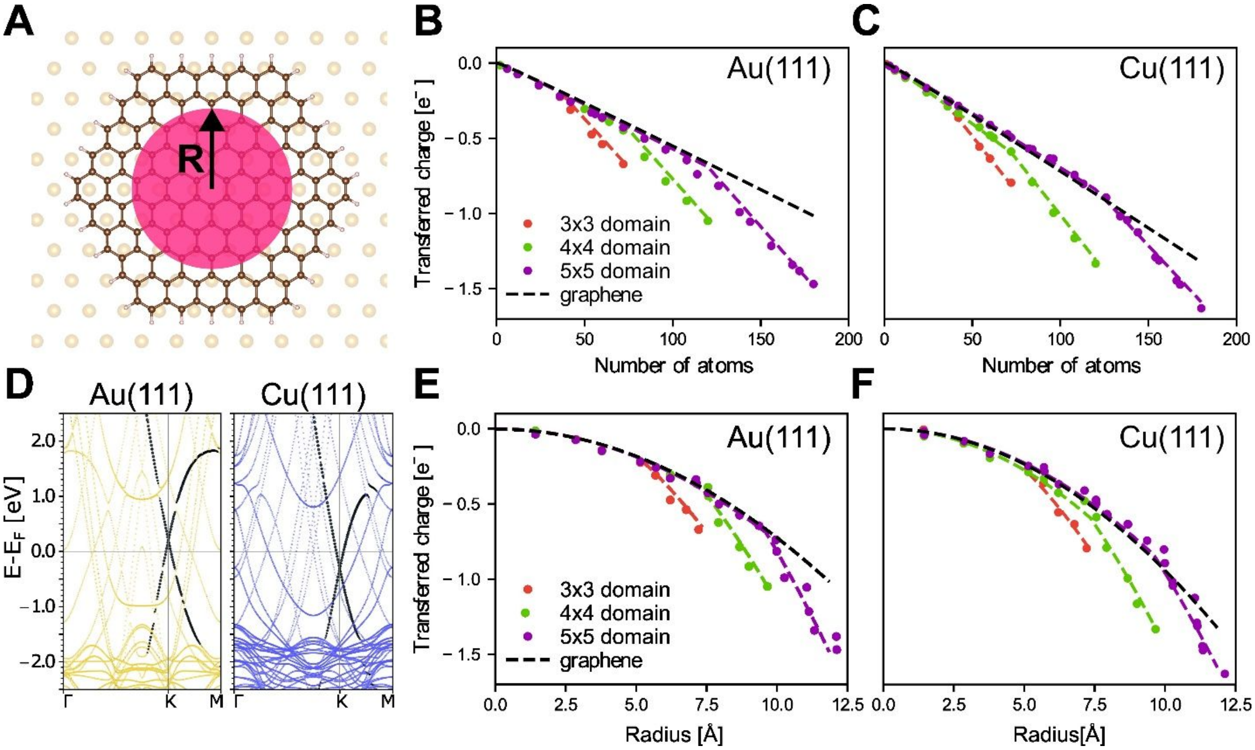

Figure S6: Analysis of the induced dipole charge distribution within the graphene domain adsorbed on the Au(111) and Cu(111) substrates. Panel **A** shows a top view of the graphene domain and the first substrate layer. The pink circle, centered on the graphene domain with a varying radius 'R' defines the area where the induced dipole charge is evaluated as a function of the number of atoms inside the circle (**B**, **C**) and the radius (**E**, **F**). Panels **B** and **E** illustrate the induced dipole charge between the graphene domains and the Au(111) substrate, while the same for Cu(111) is shown in panels **C** and **F**. Dashed colored lines in panels **B, C, E, F** represent fits from our phenomenological model and the dashed black lines show the induced dipole charge between a graphene and a respective substrate. Panel **D** shows the calculated band structure of the graphene layer adsorbed on the Au(111) substrate (left) and on the Cu(111) substrate (right). Black color indicates the projections of the calculated wave functions onto the carbon p_z orbitals.

Table S1. Calculated number of electrons transferred from the hydrogenated domains to the Au(111) and Cu(111) substrates using the modified Bader charge analysis and the cumulative charge transfer function. The relative differences of the transferred charge are below 2.4% for the Au(111) substrate and 11.2% for the Cu(111) substrate.

	Au(111)			Cu(111)		
	3×3	4×4	5×5	3×3	4×4	5×5
Modified Bader	0.67	1.04	1.47	0.80	1.33	1.63
Max(Q(z))	0.66	1.03	1.48	0.72	1.21	1.58
Relative differences (%)	2.4	1.6	-1.0	11.2	10.1	3.0

Table S2. Fitted parameters of our model to describe the charge transferred to the graphene domains with varied sizes and to the graphene sheet adsorbed on Au(111) and Cu(111) substrates: Charge density of the inner and outer regions (σ_i, σ_o) and the width of the outer region d.

	Au(111)				Cu(111)			
	3×3	4×4	5×5	graphene	3×3	4×4	5×5	graphene
σ_i (e^{-}Å^{-2})	$-2.38 \cdot 10^{-3}$			$-2.3 \cdot 10^{-3}$	$-3.5 \cdot 10^{-3}$		$-2.9 \cdot 10^{-3}$	$-3.0 \cdot 10^{-3}$
σ_o (e^{-}Å^{-2})	$-5.24 \cdot 10^{-3}$			-	$-6.0 \cdot 10^{-3}$		$-4.9 \cdot 10^{-3}$	-
d (Å)	2.25			-	2.25			-

Table S3. Calculated dipole moments induced by the interaction of the graphene domains with the Au(111) and Cu(111) substrates. Values obtained from the cumulative charge transfer function are in perfect agreement with the compensating dipole moment of the SCF run.

	Au(111)			Cu(111)		
Domain size	3×3	4×4	5×5	3×3	4×4	5×5
Cum. charge transfer function (e^{-}Å)	-1.21	-1.83	-2.45	-1.37	-2.27	-2.75
SCF dipole corrections (e^{-}Å)	-1.22	-1.83	-2.48	-1.39	-2.29	-2.75
Model (e^{-}Å)	-1.15	-1.82	-2.60	-1.37	-2.27	-2.75

Table S4. Comparison of dipole moments between the hydrogen-terminated and metal-terminated graphene domains with varying sizes upon adsorption on Au(111) and Cu(111) substrates. The resulting dipole moments for metal-terminated domains are smaller compared to the hydrogen-terminated domains.

	Au(111)			Cu(111)		
	3×3	4×4	5×5	3×3	4×4	5×5
H-terminated (e^{-}Å)	-1.22	-1.83	-2.48	-1.39	-2.29	-2.75
metal-terminated (e^{-}Å)	-0.16	-0.46	-0.89	-0.25	-0.19	-0.79

Electrostatic (repulsive) interaction. As a result of charge redistribution, interface electric dipoles develop on each graphene domain and the domain-domain interaction can be treated as dipole-dipole interaction. This interaction is repulsive (dipoles pointing in the same direction) and has been previously considered to be of key importance in the alignment of 2D materials on liquid metal catalysts.[12] Based on molecular dynamics and finite-element calculations, they have estimated the dipole moment of 1.22 D per carbon atom, which is approx. 4.1×10^{-30} C.m per carbon atom. Our calculations based on DFT give 2.9×10^{-30} C.m per carbon atom. Therefore, although we have used different approach to calculate the charge transfer, we have arrived at very similar values of the dipole moment.
We have conducted a calculation of electrostatic energy for a simplified geometry of two identical circular graphene domains floating on a flat metal surface. The electrostatic energy is calculated by integrating over four objects two graphene domains (indices 1 and 2) and two mirror charges inside the substrate (indices 3 and 4):

$$E_{el} = 2\int_1\int_2 \frac{1}{4\pi\varepsilon_0}\frac{dQ_1 dQ_2}{|\boldsymbol{r}_1 - \boldsymbol{r}_2|} + 2\int_1\int_4 \frac{1}{4\pi\varepsilon_0}\frac{dQ_1 dQ_4}{|\boldsymbol{r}_1 - \boldsymbol{r}_4|}.$$

The charge distributions which we have considered, are either constant over the graphene domain, or nonhomogeneous with highest values towards the edges (to cover both possibilities, due to uncertainty in DFT prediction for large domains). Another variable is the scaling of total charge – we have considered linear and quadratic scaling with radius. The linear scaling is reasonable for the charge concentrated at the edges, while the quadratic would be appropriate for charge uniformly distributed on the graphene surface.

Having calculated electrostatic energies, we have compared them first to thermal fluctuations $E_{thermal} = k_B T$, which may enforce attachment of otherwise repulsive domains. The distance of the domains was sought for at which the electrostatic and thermal energy match. This is then compared with the minimum distances of the domains measured from the experimental data (e.g. (Fig. 2B, last image in the sequence).

Recalling the experiments, the domains oscillate, keep certain distances from each other but do not attach to each other until a certain point. From the experimental in-situ observations we have deduced amplitudes of the wobbling motion and minimum distances between individual domains for a wide set of geometries. Interestingly, the amplitudes and minimum distances seem to be independent on the domain sizes (Fig. S16) and instead strongly scale with the width of the uncovered area between the domains (Fig. 2B). This behavior is in striking contrast with the results received from electrostatic potential calculation results. If the actual size of the domains does not matter, as is suggested by results shown in Fig. 2B and Fig. S16, and the only defining parameter is the separation of the domains, then the charge on the domain (and, hence, the electrical dipole) has to be constant, independent on the domain size, in case of dominance of electrostatic forces. This is clearly not the case.

van der Waals forces. In order to model the experimental observation, we have added an attractive van der Waals component. To estimate the magnitude of van der Waals forces which could act as the attractive counterpart of repulsive electrostatic force mentioned above, we use the formula describing the forces acting between two identical (circular) graphene domains of radii R, derived in Ref. 17:

$$F_{vdW} = \frac{-15 A_H h^2 \sqrt{R}}{512 D^{7/2}},$$

where $A_H \sim 1 \times 10^{-19}$ J is the Hamaker constant, $h = 0.34$ nm is the thickness of the graphene layer (interlayer distance in graphite) and D is the separation distance between the two domains. If we consider an example geometry for which we have observed the oscillations, that is discs of R = 200 nm, separated by distance D = 50 nm, we obtain an estimation for the magnitude of vdW forces $F_{vdW} \cong -9 \cdot 10^{-18}$ N, a significantly weaker force compared to electrostatic one. Fig. S5 shows the dependence of the calculated vdW potential on the distance between the domains, further illustrating that van der Waals forces are too weak to cause attraction of the domains at the distances observed in experiment.

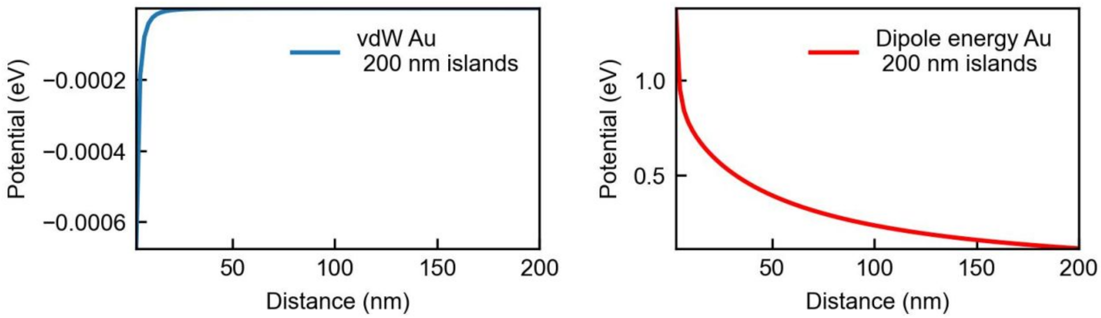

Figure S7: **Calculated potential distribution between 200 nm graphene domains on Au**. A potential-distance dependence resulting from the van der Waals interaction (left) and electrostatic interaction (right) between the domains.

Capillary forces. Previously, capillary forces have been speculated to induce a nanoscale 'Cheerios effect',[11,12] which is observed for floating macroscopic objects. However, specifically for monolayer graphene, there is no liquid meniscus around the domain, as evidenced by our in-situ measurements (Fig. 1, and Fig. S9).

Discussion of the possible presence of meniscus around graphene domains floating on a liquid metal

Below we summarize our experimental attempts to detect and characterize the morphology of the liquid metal close to the graphene domain edge. In Fig. S8 we show that the in-situ approach is necessary for any deduction on the possible meniscus presence. Next, we closely inspect in-situ secondary electron images on solid, pre-molten and liquid substrates (Fig. 9). Lastly, we show a topography map of a graphene domain on liquid gold acquired by AFM (line profile shown in Fig. 1). It should be noted that neither HT-AFM nor SEM images acquired during our experiments allow the detection of nanoscale surface undulations of the liquid metal surface or possible Ångstrom-scale bending at the graphene edge predicted by the DFT calculations, due to limited lateral resolution.

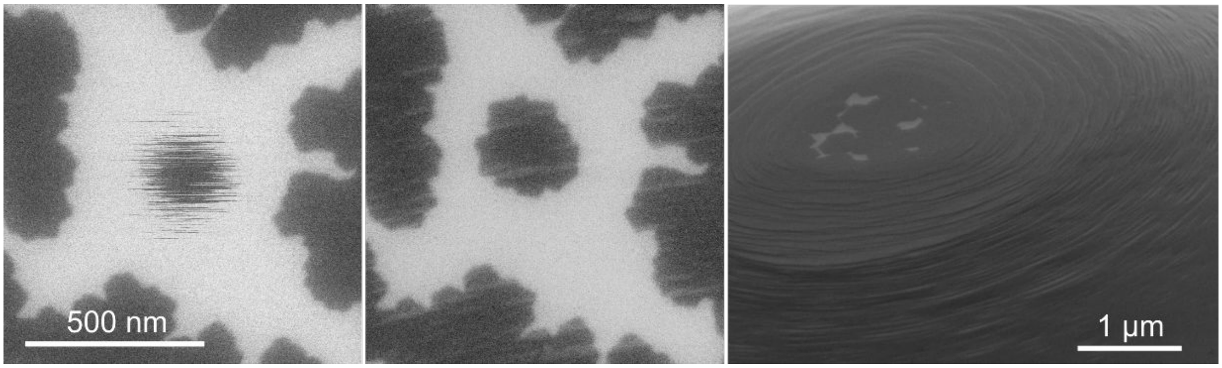

Figure S8. *In situ* SEM images from an Au sample during the CVD of graphene and after cooling down. The images reveal surface roughness emergence after solidification. Left: Undercooled liquid gold with graphene domains at 1303 K and 1 Pa ethylene, Middle: the same area just after slow cooling and solidification at 1293 K. Surface exhibits significant roughness, visible as the bright lines across the graphene domains and the substrate. Right: Au droplet that was almost fully covered with graphene in liquid state. As soon as it was cooled to 1273 K and solidified, faceting and roughness emerges (nicely visible in the secondary electron image). This behavior clearly illustrates the necessity of *in situ* investigations of the surface topography, as the *ex situ* analyses would provide misleading conclusions on the presence of meniscus. Electron beam imaging conditions: Left, middle: 10 keV, 0.8 nA, right: 20 keV, 0.4 nA.

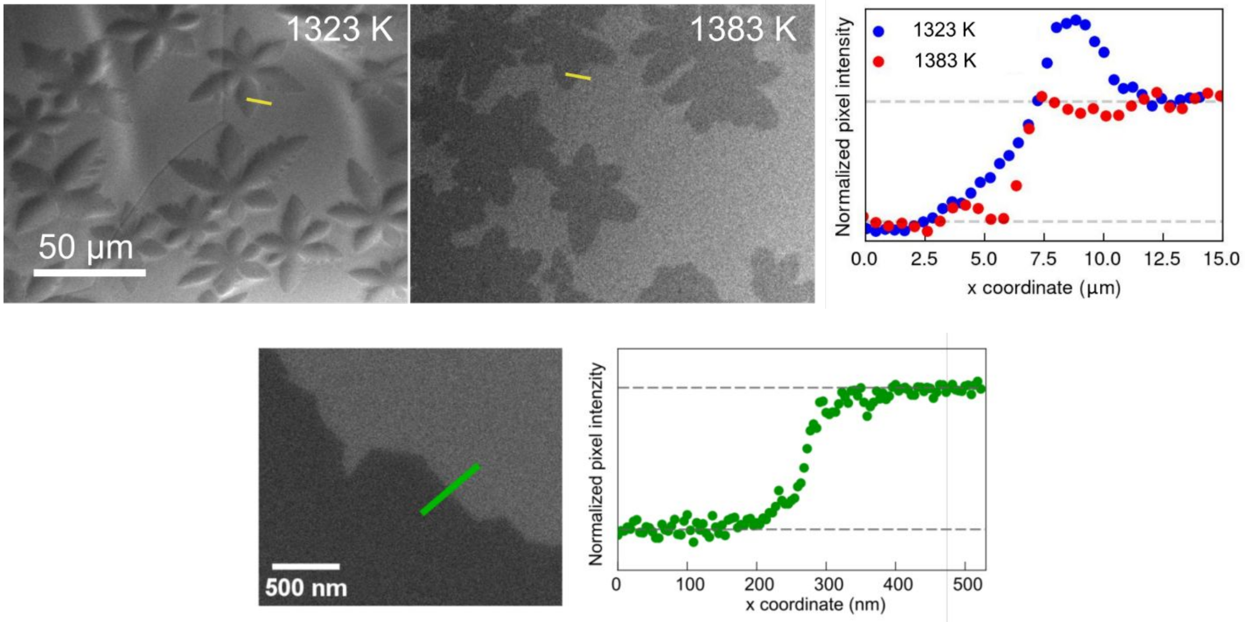

Figure S9. Representative secondary electron (SE) images of graphene domains grown on pre-molten and liquid Cu at different temperatures, and on liquid Au. First, we note that SEM images do not show any 'rim' around the graphene domain edges on solid substrates (Fig. 1). (top) In this experiment, graphene was grown with $p_{C_2H_4}$ = 1 ×10^{-2} Pa at T = 1323 K, slightly below the melting point $T_{m,\, Cu}$ of Cu, a bright rim appears around the domains. The rim disappears when the substrate is molten at T = 1383 K. Generally, secondary electrons are very sensitive to the curvature of the emitting surface (observation of single atomic steps is possible)[18], hence, any nanometer-scale irregularity of the surface is enhanced in SE image. The SE signal profiles on the right, measured along the yellow lines in the SEM images, demonstrate SE signal enhancement around the domain's edges just before melting, indicating surface swelling around the domain. Upon melting, the surface becomes flatter and the SE signal enhancement disappears. This observation supports the conclusion that no large-scale liquid meniscus forms around the graphene domains on a fully molten substrate. Electron beam imaging conditions: 5 keV, 1 nA. (bottom) Similar to Cu, graphene domain grown on liquid gold ($p_{C_2H_4}$ = 30 Pa at T = 1363 K) does not exhibit any meniscus. Electron beam imaging conditions: 10 keV, 200 pA.

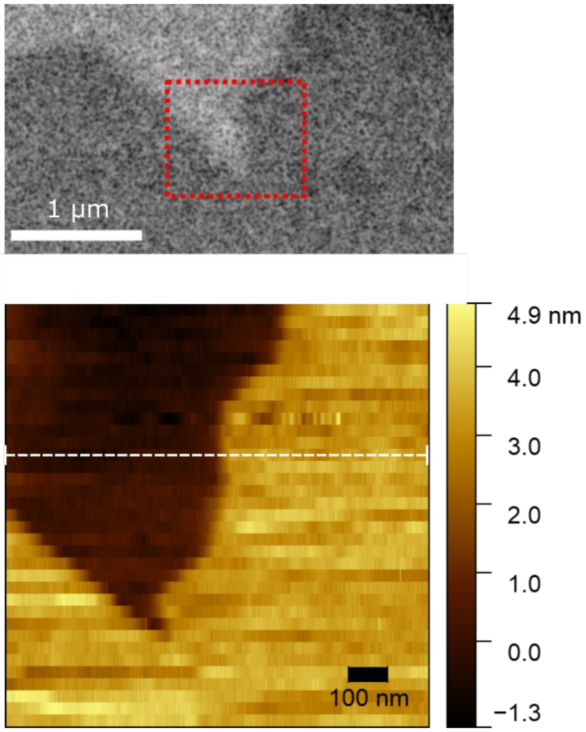

Figure S10. SEM image (top) and HT-AFM topography map (bottom) taken at the area marked by red dotted square in SEM image (1343 K, graphene on a liquid gold). To minimize possible image disturbance, the graphene growth was terminated before the AFM imaging took place. Additionally, graphene domain was not freely floating on the liquid surface; instead, it was pinned to a stable bunch of other graphene domains. The line profile in Fig. 1 was taken along the white dashed line, as depicted in the figure. The map has been processed by row alignment procedure and levelled by a subtraction of a sphere. The area of liquid gold was used for the row alignment (therefore gold has the same height in all rows). The z-axis has been shifted to set the mean level of liquid gold to 0 nm. Note that the measured step height is dependent on the instrumental settings[19] and, therefore, not realistic. The AFM measurement is thus used solely to dismiss the possible meniscus presence.

Supplemental experiments for discussion of wobbling

The rapid wobbling of domains may occur due to a variety of reasons (gas or liquid flow, electron beam effect etc.). The experiments shown below serve to support our conclusions that the behavior of the domains observed in experiments is explained only by the existence of capillary fluctuations.

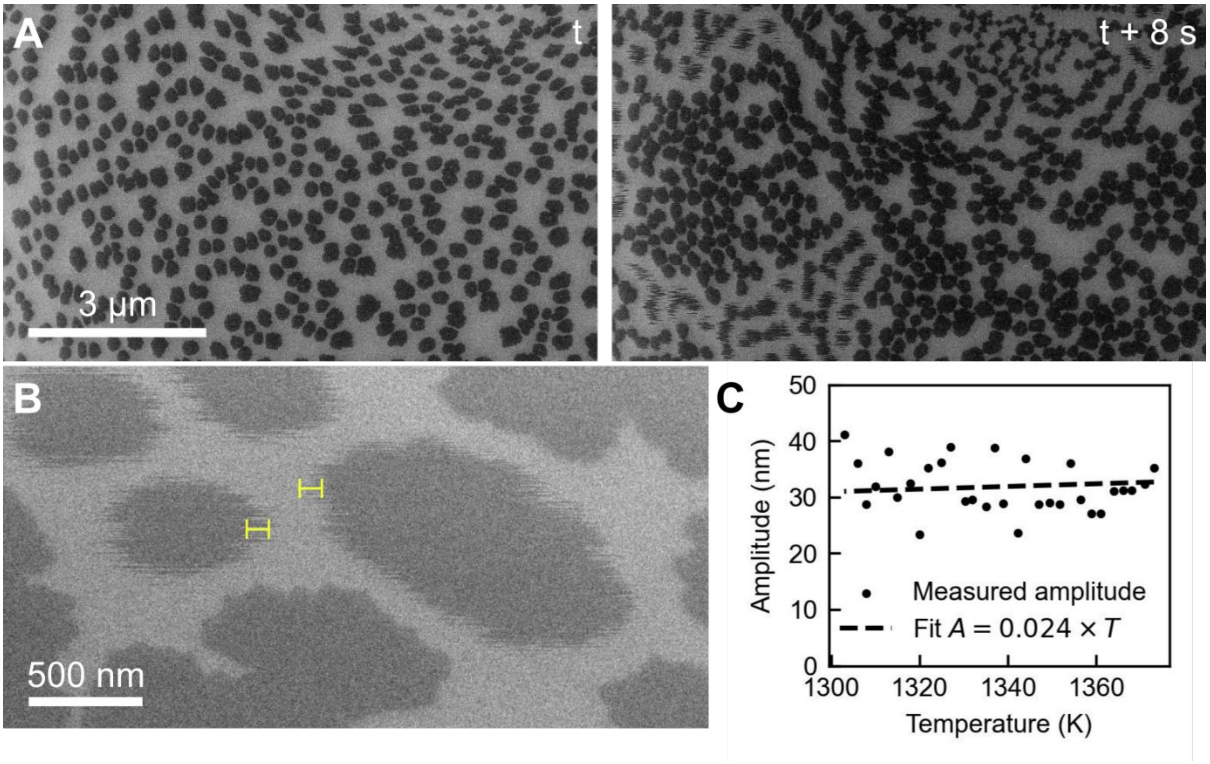

Figure S11. Effect of temperature and domain size on domain dynamics: *In situ* SEM images acquired from Au sample during the CVD of graphene using 35 Pa of ethylene. In panel **A**, the graphene domains were nucleated on solid Au at 1223 K (left) and then Au rapidly melted and undercooled back to 1223 K (right, after 8 s). Many domains immediately cluster together, and these agglomerates are pinned to Au droplet edges. Those domains left floating are oscillating on a liquid surface. A careful inspection of the figure reveals relatively large floating domains that oscillate as well; this is clearly demonstrated in panel **B**. **(C)** Amplitude of the oscillations shows very weak dependence on temperature within the accessible temperature range. These data also support Fig. 2B, as they clearly show that the oscillations are not correlated to any other system variable (e.g., domain size) up to the resolution limit. Electron beam imaging conditions: 10 keV, 0.4 nA (**A**), 10 keV, 0.8 nA (**B**).

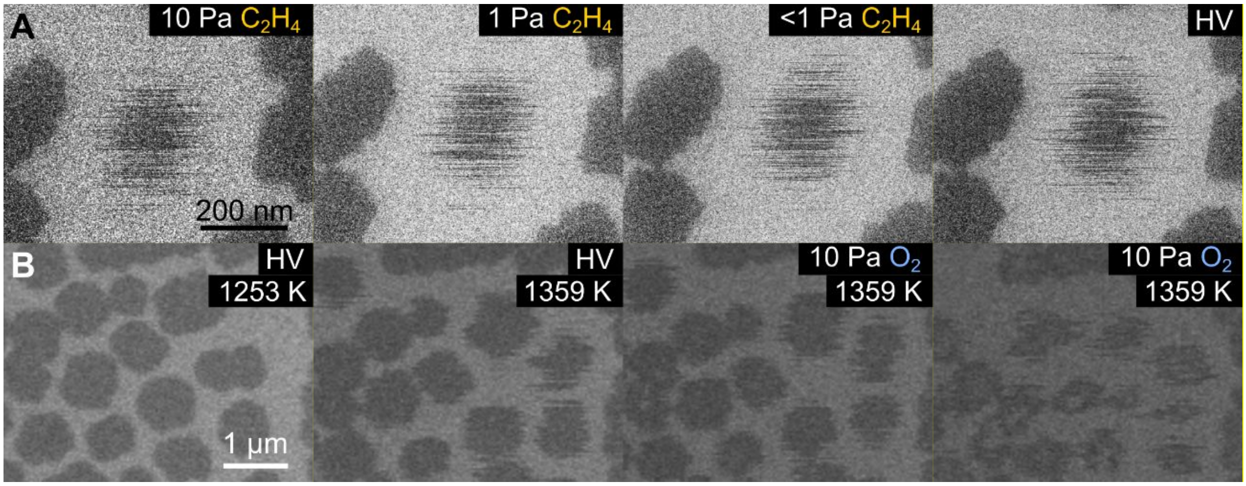

Figure S12. Effect of the deposition flux on domain dynamics: *In situ* SEM images from an Au sample during the CVD of graphene at different partial pressures of ethylene. The domain oscillations are independent of pressure. In panel **A**, the sample temperature is 1353 K, the pressure is noted in the images. HV = high vacuum (10^{-3} Pa). In panel **B**, the first image is obtained for solid Au at 1253 K, while the subsequent frames are acquired at 1359 K. The domain oscillations are present also in O_2 gas, which results in etching of the domains. That is, the domains keep oscillating during graphene growth, in vacuum, and during etching as well. Electron beam imaging conditions: 10 keV, 0.4 nA (**A**), 10 keV, 0.2 nA (**B**).

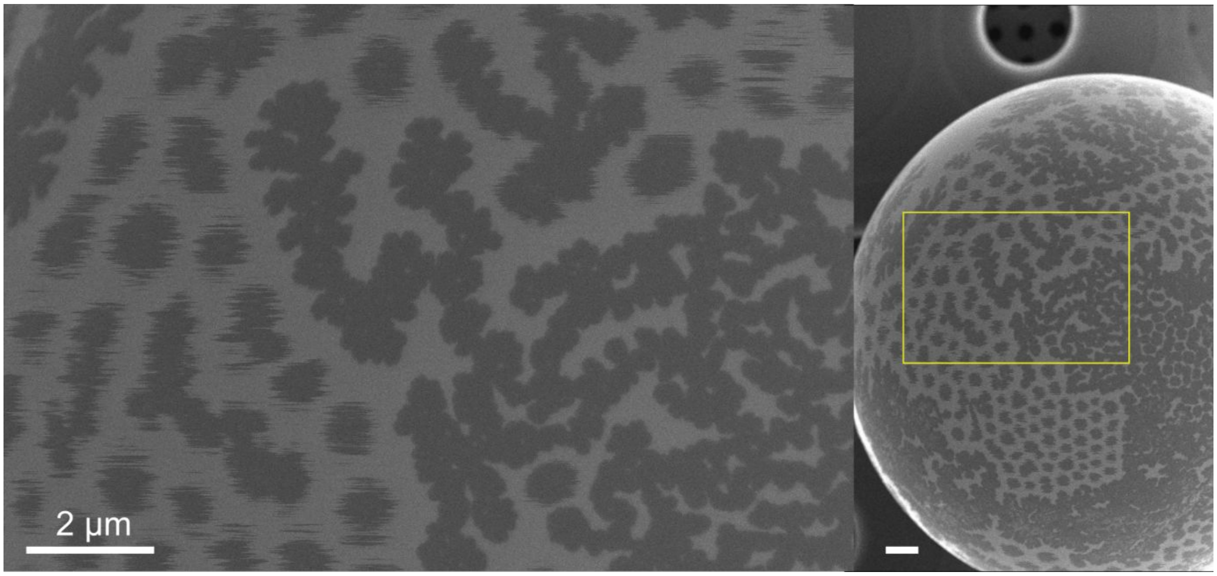

Figure S13. Effect of electron beam on domain dynamics: *In situ* SEM images acquired from Au sample during the CVD of graphene at an ethylene pressure of 15 Pa and 1311 K. SEM imaging conditions: 10 keV, 0.2 nA, dwell time 5 μs, electron beam dose rates: (left) 0.17 pA/nm^2 and (right) 0.01 pA/nm^2. We were imaging the yellow framed area for about 300 s (5 minutes) and observed the floating domain oscillations. The right image is a 4× demagnified image under the same beam conditions (hence, 16× lower electron dose), we see previously unexposed areas where floating domains exhibit identical oscillation behavior as in the previous scanning window. The oscillations, however, occur under different beam conditions and the domains behave similarly even if the beam is scanning a different area. Moreover, imaging under different beam conditions (including also a different microscope) does not affect the data plotted in Fig. 2B, where all the curves fall onto each other despite being taken under different beam conditions.

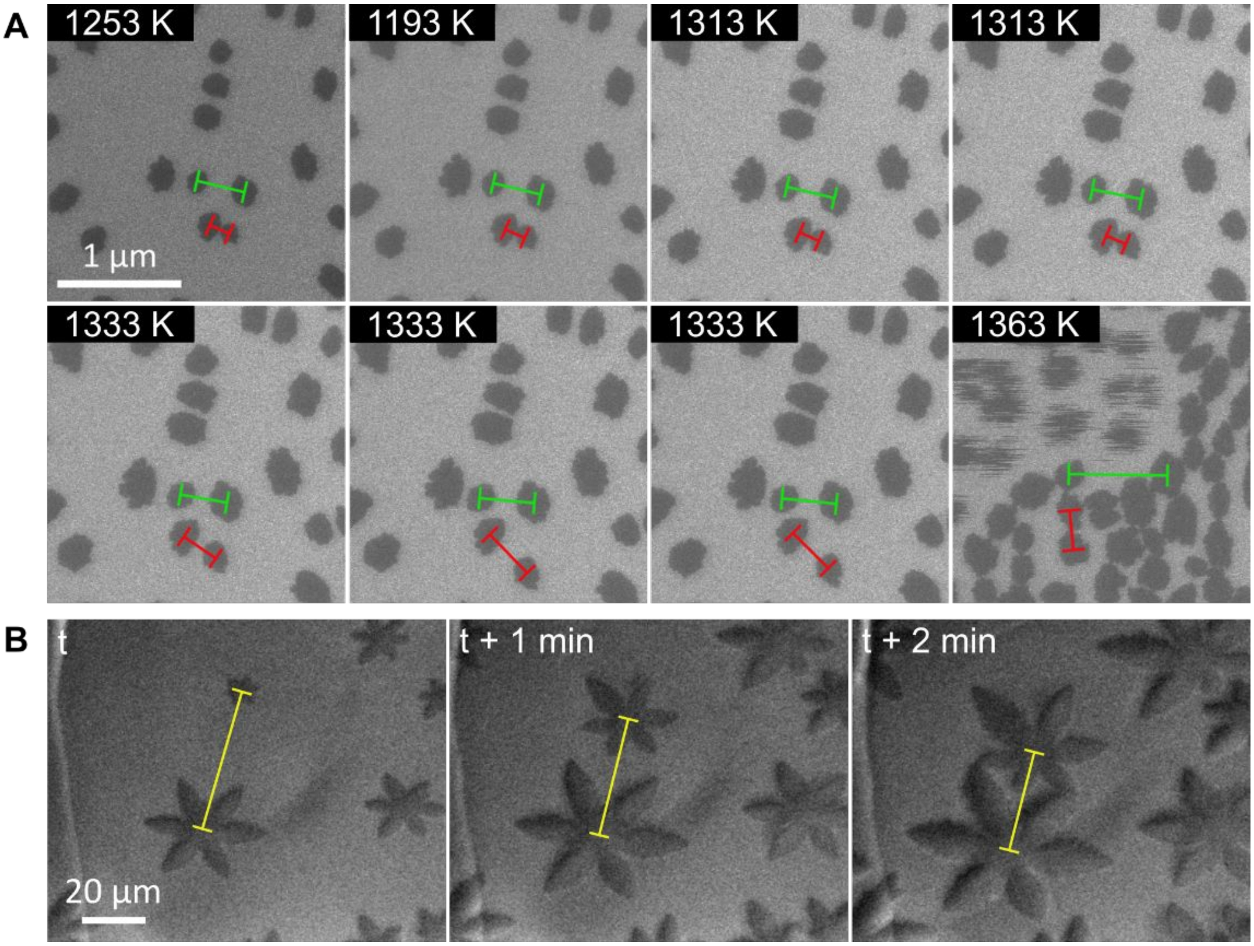

Figure S14. Effect of the state of the substrate on domain dynamics and wobbling: *In situ* SEM image sequence in panel **A** was taken during heating of the Au sample with graphene domains. The domains remain pinned to the substrate at lower temperatures. Slightly below the melting point the domains start to move (1333 K), but wobbling is not observed. After substrate melting (1363 K, last image), the domains partially coalesce and the rest starts to wobble. Similar behavior is observed also on Cu (**B**). The colored lines mark the distances between the domains. Experimental conditions: Au substrate, ethylene pressure 30 Pa, electron beam: 10 keV, 0.4 nA (**A**); Cu substrate, T = 1343 K, ethylene pressure 2 $\times 10^{-2}$ Pa, electron beam: 5 keV, 1 nA (**B**).

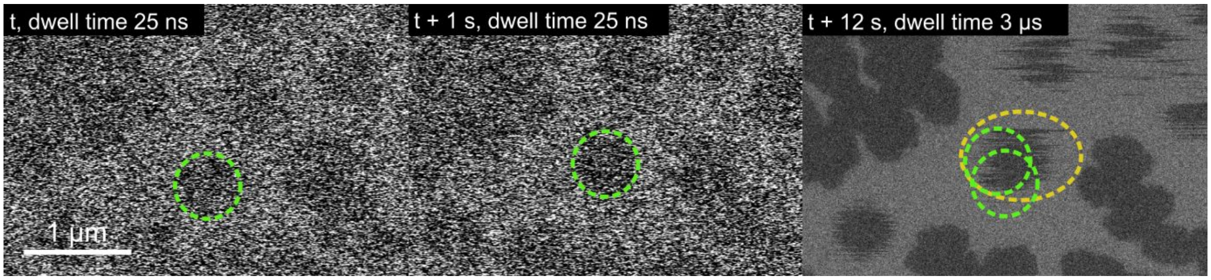

Figure S15. Effect of dwell time on domain dynamics: *In situ* SEM images of an undercooled Au sample during the CVD of graphene acquired using different dwell times. Imaging of unattached graphene islands on undercooled gold at different dwell times reveals that the oscillations seen in SEM images are related to domain movements, not to changes in shape (apart from increasing in size) – center of mass (deduced from the short dwell time image, green circles) falls into the envelope (yellow ellipse) of oscillations measured on the larger dwell time image. Beam energy 10 keV, current 0.8 nA, temperature 1223 K, ethylene pressure 10 Pa.

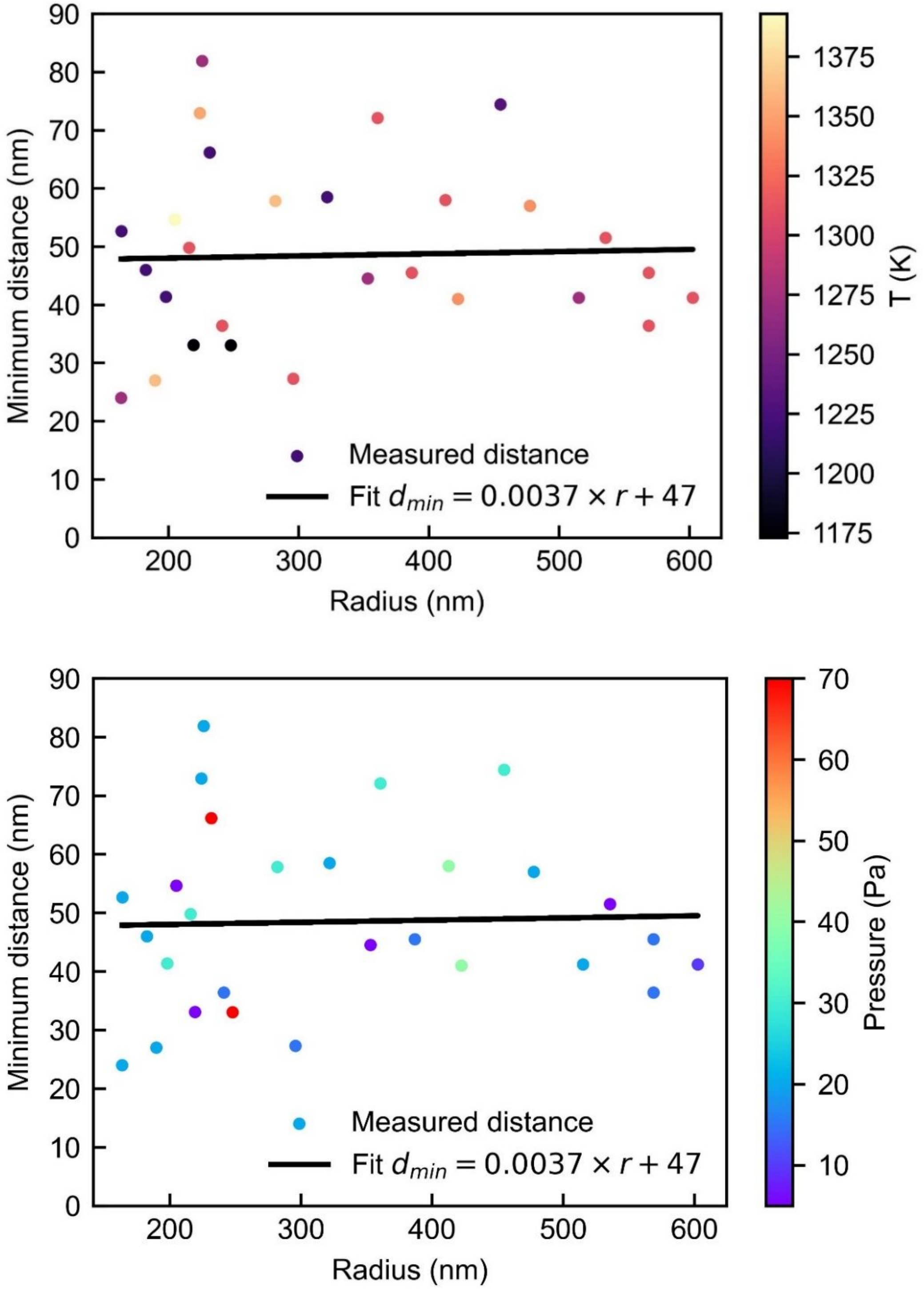

Figure S16. Analysis of the critical distance as measured on liquid gold for a variety of experimental conditions (1223 – 1373 K, 5-70 Pa ethylene) and domain sizes. Only a very weak dependence of the minimum distance on the sizes of domains is seen. Each datapoint represents a different experiment, the color code stands for the sample temperature (top) or C_2H_4 pressure (bottom). No correlation is found between the minimum distance and experimental conditions.

Discussion of attractive interaction in between the domains mediated by the capillary waves

The floating domains are attracted to each other on mesoscale distances. This behavior is observed in experiments here (see Fig. S17 below) and elsewhere.[12,20] Although this phenomenon was previously ascribed to a capillary force, our modelling clearly shows that capillary undulations induce this effect as well (see Movie S10) - attractive forces in between the domains (blue arrows) arise from the fact that there are much more available capillary waves (with different wave vectors) on the outer (free surface) side of the domains.

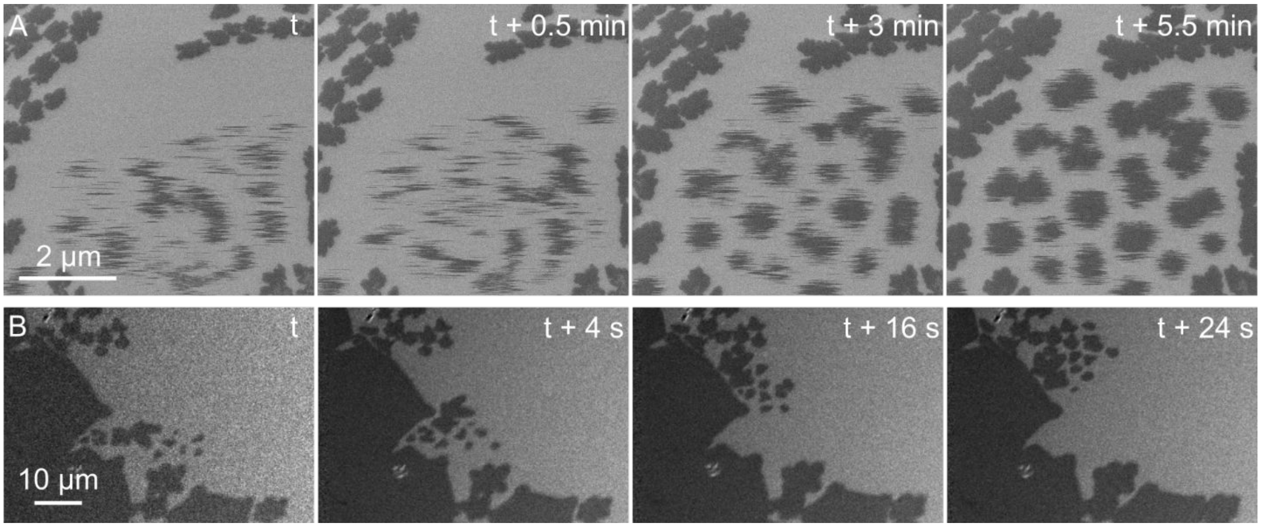

Figure S17. Observation of multiple domains within a large uncovered area on an already stabilized liquid Au (A) and Cu (B) surface. Initially, at low coverage, the domains do not occupy the maximum available space, but tend to cluster together and assemble into a semi-regular pattern, still maintaining similar average distances. Similar behavior is seen in Movie S10. Additionally, in panel **B**, there is a preferential movement of the group of domains along the compartment edge. The domains remain in the cluster during the movement. As the model implies (see below), this behavior is a consequence of a different number of surface waves on each side of a domain (i.e., a domain is attracted to the closest object nearby). The experimental conditions: (**A**) Au, 1273 K, 20 Pa ethylene, electron beam: 10 keV, 0.8 nA, (**B**) Cu, 1373 K, 1.2 $\times 10^{-2}$ Pa, electron beam: 5 keV, 1 nA..

Description of supplementary movies

Movie S1: Full movie of experimental workflow.
In situ scanning electron microscopy (SEM) movie (10 keV, 800 pA) acquired at 5 frames/s during graphene growth at the temperature $T = 1223$ K and ethylene pressure $p_{C2H4} = 30$ Pa. At $t = 368\ s$, the temperature is rapidly changed to $T = 1337$ K and undercooled back to $T = 1223$ K within one frame by rapidly altering the heating power. The change from the static graphene domains to either interconnected or oscillating takes place within 50 lines during the scan, corresponding to a real time of 0.8 s. An approximate lateral growth rate 0.5 nm/s was observed before the melting of the substrate.

Movie S2: Growth of graphene domains on solid gold.
In situ SEM movie (20 keV, 400 pA, 7 frames/s) of graphene domains on solid Au during the deposition of graphene at temperature $T = 1223$ K using ethylene partial pressure $p_{C_2H_4} = 30$ Pa.; The video time t_{video} =526 s.

Movie S3: Alignment of graphene domain arrays on liquid gold.
In situ SEM movie (20 keV, 400 pA, 7 frames/s) of the same graphene domains from Movie S2 on molten Au during the deposition of graphene at temperature $T = 1373$ K using ethylene partial pressure $p_{C_2H_4} = 30$ Pa.; The video time t_{video} =188 s.

Movie S4: Rotating graphene domain in an enclosed space. *In situ* SEM movie (5 keV, 1 nA, 7 frames/s) of molten Cu during graphene growth at $T = 1373$ K with $p_{C2H4} = 1.2{\cdot}10^{-2}$ Pa, t_{video} =140 s. The figure below resulted from a frame-by-frame analysis of the movie, which allows to determine the rotational motion of the domain from those frames where the domain shape is undistorted (four snapshots on the right, with a mirror axis highlighted by the dashed line). The graph on the left shows a full analysis of rotational movement of the domain during observation. The envelope (dashed line in the graph) clearly points to 5 degrees, parallel with the edges of the surrounding compartment. The angle of the rotating domain with respect to x-axis was determined utilizing a major axis of an ellipse fitted to the segmented domain.

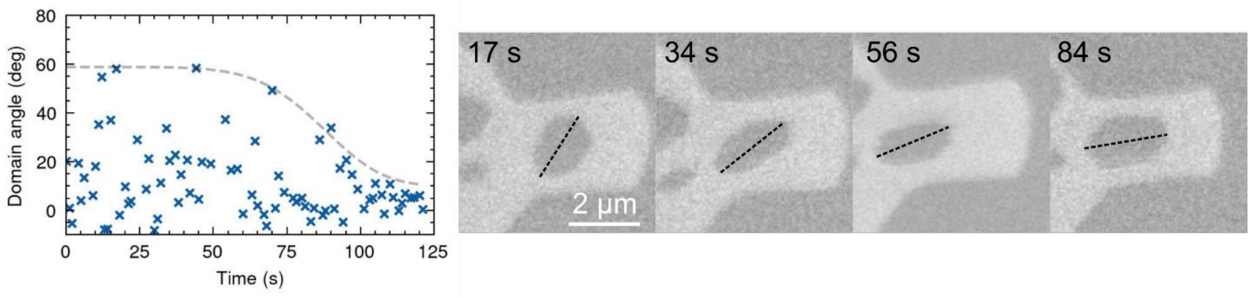

Movie S5: A graphene domain enclosed inside a compartment. *In situ* SEM movie (10 keV, 400 pA, 7 frames/s) from liquid Au at $T = 1313$ K with $p_{C_2H_4} = 15$ Pa showing wobbling of a graphene domain fully enclosed within a region bounded by other domains. $t_{video} = 23.75$ min.

Movie S6: Simulation of a domain behaviour on a liquid. A simulated movie showing a growing two-dimensional (2D) hexagonal domain floating on a liquid within a hexagonal compartment (see figure below, left). The dark blue and yellow colors denote low and high amplitudes, respectively, of standing waves formed on the liquid surface. The short red lines indicate forces acting on the domain edges; blue arrow represents magnitude and direction of the total force on the domain. (Center) Plot of the domain position (x,y) and rotation (rot). (Right) Plot of F_x and F_y, the *x*- and *y*-components respectively of the total force and the momentum M_z acting on the domain.

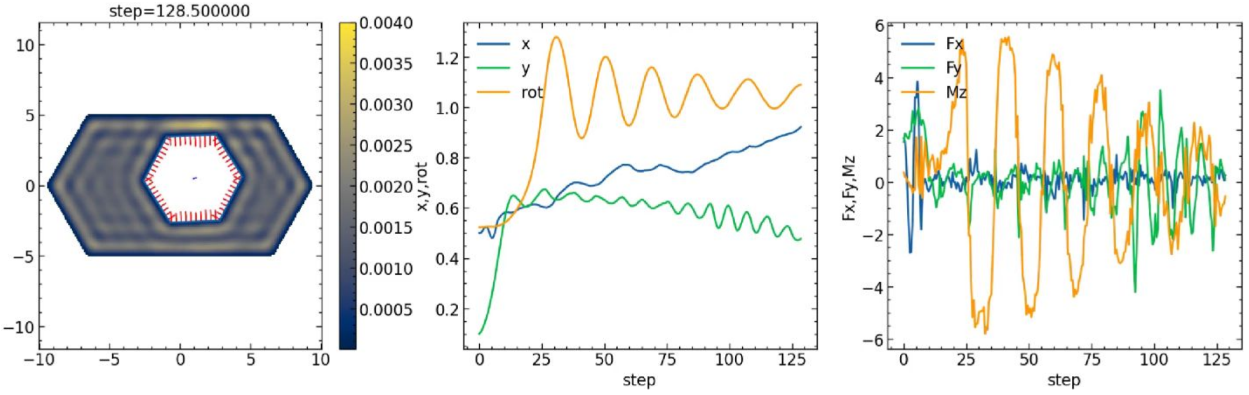

Movie S7: Simulation of a domain behavior on a liquid with different viscosity. Simulated movie showing wobbling of a domain calculated using the same parameters as in Movie S6 but with lower viscosity (damping set to one-half of that in Movie S6).

Movie S8: Domain assembly inside a graphene compartment on liquid Cu. *In situ* SEM movie (1 keV, 1 nA, 7 frames/s) during graphene growth on liquid Cu at T = 1373 K with $p_{C_2H_4}$ = 8 × 10^{-3} Pa. t_{video} = 231 s.

Movie S9: Assembly of multiple interacting domains. A simulated movie showing evolution of six growing domains enclosed within an extended hexagonal space. Initially randomly placed and oriented domains (see figure below, left) spontaneously assemble into a nearly regular array (middle) as a result of surface undulations. With increasing size, the domains attach (right), similar to the experiment shown in Fig. 1B (Movie S3).

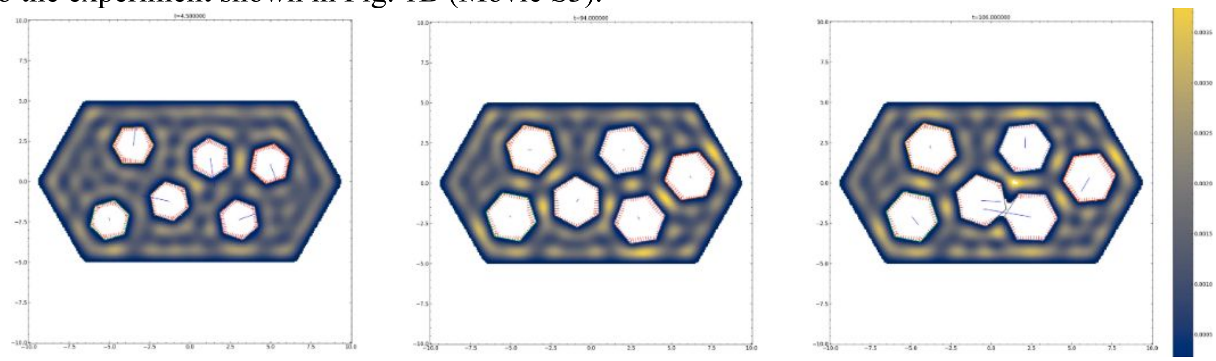

Movie S10: An attractive force between the floating domains. A simulated movie showing the evolution of several domains enclosed within a large hexagonal space. For simplicity, the domain sizes are held constant in this simulation. Although there is plenty of free space within the compartment, the domains remain close to each other at all times.

References

(1) Nemes-Incze, P.; Osváth, Z.; Kamarás, K.; Biró, L. P. Anomalies in Thickness Measurements of Graphene and Few Layer Graphite Crystals by Tapping Mode Atomic Force Microscopy. *Carbon N Y* **2008**, *46* (11), 1435–1442. https://doi.org/10.1016/j.carbon.2008.06.022.

(2) Kresse, G.; Hafner, J. *Ab Initio* Molecular Dynamics for Liquid Metals. *Phys Rev B* **1993**, *47* (1), 558–561. https://doi.org/10.1103/PhysRevB.47.558.

(3) Kresse, G.; Joubert, D. From Ultrasoft Pseudopotentials to the Projector Augmented-Wave Method. *Phys Rev B* **1999**, *59* (3), 1758–1775. https://doi.org/10.1103/PhysRevB.59.1758.

(4) Grimme, S.; Antony, J.; Ehrlich, S.; Krieg, H. A Consistent and Accurate *Ab Initio* Parametrization of Density Functional Dispersion Correction (DFT-D) for the 94 Elements H-Pu. *J Chem Phys* **2010**, *132* (15), 154104. https://doi.org/10.1063/1.3382344.

(5) Monkhorst, H. J.; Pack, J. D. Special Points for Brillouin-Zone Integrations. *Phys Rev B* **1976**, *13* (12), 5188–5192. https://doi.org/10.1103/PhysRevB.13.5188.

(6) Khomyakov, P. A.; Giovannetti, G.; Rusu, P. C.; Brocks, G.; Van Den Brink, J.; Kelly, P. J. First-Principles Study of the Interaction and Charge Transfer between Graphene and Metals. *Phys Rev B Condens Matter Mater Phys* **2009**, *79* (19), 195425. https://doi.org/10.1103/PhysRevB.79.195425.

(7) Henkelman, G.; Arnaldsson, A.; Jónsson, H. A Fast and Robust Algorithm for Bader Decomposition of Charge Density. *Comput Mater Sci* **2006**, *36* (3), 354–360. https://doi.org/10.1016/j.commatsci.2005.04.010.

(8) Tostmann, H.; DiMasi, E.; Pershan, P. S.; Ocko, B. M.; Shpyrko, O. G.; Deutsch, M. Surface Structure of Liquid Metals and the Effect of Capillary Waves: X-Ray Studies on Liquid Indium. *Phys Rev B* **1999**, *59* (2), 783–791. https://doi.org/10.1103/PhysRevB.59.783.

(9) Sanyal, M. K.; Sinha, S. K.; Huang, K. G.; Ocko, B. M. X-Ray-Scattering Study of Capillary-Wave Fluctuations at a Liquid Surface. *Phys Rev Lett* **1991**, *66* (5), 628. https://doi.org/10.1103/PhysRevLett.66.628.

(10) Zhang, Y.; Sprittles, J. E.; Lockerby, D. A. Thermal Capillary Wave Growth and Surface Roughening of Nanoscale Liquid Films. *J Fluid Mech* **2021**, *915*, A135. https://doi.org/10.1017/jfm.2021.164.

(11) Vella, D.; Mahadevan, L. The “Cheerios Effect.” *Am J Phys* **2005**, *73* (9), 817–825. https://doi.org/10.1119/1.1898523.

(12) Jankowski, M.; Saedi, M.; La Porta, F.; Manikas, A. C.; Tsakonas, C.; Cingolani, J. S.; Andersen, M.; De Voogd, M.; Van Baarle, G. J. C.; Reuter, K.; Galiotis, C.; Renaud, G.; Konovalov, O. V.; Groot, I. M. N. Real-Time Multiscale Monitoring and Tailoring of Graphene Growth on Liquid Copper. *ACS Nano* **2021**, *15* (6), 9638–9648. https://doi.org/10.1021/acsnano.0c10377.

(13) Gong, C.; Lee, G.; Shan, B.; Vogel, E. M.; Wallace, R. M.; Cho, K. First-Principles Study of Metal-Graphene Interfaces. *J Appl Phys* **2010**, *108* (12), 123711. https://doi.org/10.1063/1.3524232.

(14) Gong, C.; Hinojos, D.; Wang, W.; Nijem, N.; Shan, B.; Wallace, R. M.; Cho, K.; Chabal, Y. J. Metal-Graphene-Metal Sandwich Contacts for Enhanced Interface Bonding and Work Function Control. *ACS Nano* **2012**, *6* (6), 5381–5387. https://doi.org/10.1021/nn301241p.

(15) Tesch, J.; Leicht, P.; Blumenschein, F.; Gragnaniello, L.; Fonin, M.; Marsoner Steinkasserer, L. E.; Paulus, B.; Voloshina, E.; Dedkov, Y. Structural and Electronic Properties of Graphene Nanoflakes on Au(111) and Ag(111). *Sci Rep* **2016**, *6* (1), 23439. https://doi.org/10.1038/srep23439.

(16) Roth, S.; Gatti, G.; Crepaldi, A.; Grioni, M. The Growth and Band Structure of a Graphene-Encapsulated Two-Dimensional Nodal Line Semimetal: Cu_2Si. *Electronic Structure* **2019**, *1* (1), 014001. https://doi.org/10.1088/2516-1075/ab02a3.

(17) Goggin, D. M.; Samaniuk, J. R. 2D Colloids: Size- and Shape-Controlled 2D Materials at Fluid–Fluid Interfaces. *Langmuir* **2021**, *37* (48), 14157–14166. https://doi.org/10.1021/acs.langmuir.1c02418.

(18) Homma, Y.; Tomita, M.; Hayashi, T. Atomic Step Imaging on Silicon Surfaces by Scanning Electron Microscopy. *Ultramicroscopy* **1993**, *52* (2), 187–192. https://doi.org/10.1016/0304-3991(93)90189-5.

(19) Nemes-Incze, P.; Osváth, Z.; Kamarás, K.; Biró, L. P. Anomalies in Thickness Measurements of Graphene and Few Layer Graphite Crystals by Tapping Mode Atomic Force Microscopy. *Carbon N Y* **2008**, *46* (11), 1435–1442. https://doi.org/10.1016/j.carbon.2008.06.022.

(20) Rein, V.; Gao, H.; Heenen, H. H.; Sghaier, W.; Manikas, A. C.; Tsakonas, C.; Saedi, M.; Margraf, J. T.; Galiotis, C.; Renaud, G.; Konovalov, O. V.; Groot, I. M. N.; Reuter, K.; Jankowski, M. *Operando* Characterization and Molecular Simulations Reveal the Growth Kinetics of Graphene on Liquid Copper During Chemical Vapor Deposition. *ACS Nano* **2024**, *18* (19), 12503–12511. https://doi.org/10.1021/acsnano.4c02070.
