# Supplementary material for: Capillary Wave Driven Dynamics of Graphene Domains during Growth on Molten Metals
Source: J Phys Chem Lett. 2025 Sep 17;16(38):10020–6. doi: 10.1021/acs.jpclett.5c02321 (PMC12478858; doi:10.1021/acs.jpclett.5c02321)
Supplement: Supplementary file 12 [file jz5c02321_si_012.pdf]

jz-2025-023212.R1

Name: Peer Review Information for "Capillary wave driven dynamics of graphene domains during growth on molten metals"

First Round of Reviewer Comments

Reviewer: 1

Comments to the Author

jz-2025-023212

Capillary wave driven dynamics of graphene domains during growth on molten metals

The manuscript presents a compelling and well-executed study on the rheotaxy of graphene on molten metal surfaces, offering valuable insights into the self-assembly mechanisms of two-dimensional materials. The authors employ a strong combination of in situ microscopy, high-temperature AFM, density functional theory, and continuum modelling to elucidate the translational and rotational motions of graphene domains during growth on molten gold and copper. The work is methodologically sound, the findings are novel, and the results are clearly presented. I consider this paper very interesting and suitable for publication after the following minor revisions:

1. The authors should discuss possible alternatives to CVD techniques for the fabrication of graphene films, particularly approaches that could avoid polydomain formation.

2. References for the melting temperatures of the metal substrates should be provided to enhance completeness and reproducibility.

3. The experimental conditions of the SEM imaging in Figure 1 should be stated explicitly. It is assumed that a BSE detector was used, but confirmation and relevant parameters would be helpful.

4. More quantitative details or references regarding the relationship between the damping constant, viscosity of the molten metals, and the observed differences in domain attachment behavior between liquid copper and liquid gold should be included to substantiate the explanation for faster attachment on low-viscosity substrates.

5. Since the proposed model neglects electric dipole interactions - which may become significant for larger domains - the authors should briefly discuss how including these effects might alter their predictions.

Best regards.

Reviewer: 2

Comments to the Author

The article entitled 'Capillary wave driven dynamics of graphene domains during

growth on molten metals' directly observes the translational, rotational, and oscillatory dynamics of graphene domains on a molten metal (Au/Cu) surface using in situ SEM, experimentally confirming the physical mechanism of capillary wave-driven self-assembly of two-dimensional materials. Furthermore, a continuum model is developed to couple capillary wave fluctuations with domain dynamics, successfully predicting oscillation, rotation, and critical adhesion behaviors. This paper provides a new paradigm for rheoepitaxy. However, the authors need to address the following issues before considering publication.

1. The authors claim that capillary waves (induced by thermal fluctuations) are the physical essence of self-assembly of graphene domains on molten metals. How can the influence of thermal gradient forces/Marangoni effects be ruled out? What about short-range electrostatics? In short, how to verify that capillary waves are the dominant factor?

2. In the AFM image in Figure 1e, why is the height of graphene is 3 nm, while the typical monolayer thickness is 0.34 nm?

3. Figure 2b shows that amplitude (A) decays nonlinearly with (R-r), but why do the data for domains of different sizes almost overlap? Does the amplitude (A) depend only on the domain distance (R-r)?

4. Figure 2b shows that the experimentally observed the critical distance of gold (50 ± 20 nm) is less than that of copper (140 ± 60 nm). Stokes' law predicts that high-viscosity media have greater damping and domain motion should stop earlier, and gold with its higher viscosity (Described in the article as 1.35× larger), should have a larger critical distance. Please explain.

5. In Figure 3a, the domains rotate while growing from a circular to a hexagonal shape. Is the reorientation of domains related to the electrostatic forces on the carbon atoms at the edge of the sample (electrostatic torque)? The model assumes perfect hexagons (Figure 3b), but the experimental domain edges are irregular (jagged in Figure 3a). Does the actual edge roughness significantly alter the capillary wave force field distribution? Is hexagonal symmetry the key and growth process dominated?

6. Does high-energy electron beam irradiation induce local thermal gradient? Electron beam perturbations (e.g., low beam current contrast) or thermal gradient effects have not been ruled out. How to reduce the influence of electron beam voltage and current in experiments?

Author's Response to Peer Review Comments:

Reply to reviewers

Below please find point-to-point response to the reviewers' comments. For clarity, we used **bold font** for the referee comments and italics for our responses, blue color for the revisions/new text, ~~crossed~~ for deleted text.

Reviewer: 1

Recommendation: This paper is publishable subject to minor revisions noted. Further review is not needed.

We sincerely appreciate the referee's recommendation to publish without further review.

The manuscript presents a compelling and well-executed study on the rheotaxy of graphene on molten metal surfaces, offering valuable insights into the self-assembly mechanisms of two-dimensional materials. The authors employ a strong combination of in situ microscopy, high-temperature AFM, density functional theory, and continuum modelling to elucidate the translational and rotational motions of graphene domains during growth on molten gold and copper. The work is methodologically sound, the findings are novel, and the results are clearly presented. I consider this paper very interesting and suitable for publication after the following minor revisions:

We thank the referee for detailed review of our work and for recognizing its significance for the community and readership of JPCL.

1. **The authors should discuss possible alternatives to CVD techniques for the fabrication of graphene films, particularly approaches that could avoid polydomain formation.**

In response to this note, we have added two additional references, modified and rearranged the introductory part of the manuscript as follows:

"Since the discovery of graphene, considerable efforts have been aimed at the production of large-area graphene sheets [https://doi.org/10.1021/acs.chemrev.0c01191]. Among the several different synthesis approaches proposed to date, chemical vapor deposition (CVD) has been the most promising method for obtaining high-quality large-area layers of graphene. ~~Using singlecrystalline substrates and by the optimal choice of growth parameters, large-domain and singlecrystalline graphene layers have been obtained~~[1–5]. CVD onto polycrystalline foils and amorphous substrates, however, typically yield polydomain graphene, i.e. with multiple rotational domains separated by boundaries; such graphene layers are undesirable for most applications. A prominent strategy to grow a large-scale single-domain graphene (and other 2D materials) has been to use single-crystalline substrates and by the optimal choice of growth parameters, large-domain and single-crystalline graphene layers have been obtained by stitching many unidirectionally aligned domains. [1–5] Another successful approach has been to form a single nucleus on the substrate and let it grow into a single-domain layer under wellcontrolled growth conditions [https://doi.org/10.1038/nmat4477]. Recently, rheotaxy…"

2. **References for the melting temperatures of the metal substrates should be provided to enhance completeness and reproducibility.**

We refer now the melting temperatures more accurately, referencing an established table [James, A. M.; Lord, M. P. Macmillan's Chemical and Physical Data; Macmillan: London, U.K., 1992]

3. **The experimental conditions of the SEM imaging in Figure 1 should be stated explicitly. It is assumed that a BSE detector was used, but confirmation and relevant parameters would be helpful.**

We agree that the acquisition conditions for each image/movie should be stated. All of the in situ SEM data are collected using secondary electrons. Hence, we made following changes:

The experimental part in the main text now contains following sentence: "All images and movies presented here were acquired using secondary electrons; beam conditions are stated in movie captions."

Because the images in the main article are extracted from movies, each movie caption states the imaging conditions.

4. **More quantitative details or references regarding the relationship between the damping constant, viscosity of the molten metals, and the observed differences in domain attachment behavior between liquid copper and liquid gold should be included to substantiate the explanation for faster attachment on low-viscosity substrates.**

We thank the referee for this note. In our model, we introduced the damping coefficient that accounts for the viscosity, as well as for the density of the substrate material, which affects the dispersion relation of the capillary waves. However, the exact formalism linking the damping coefficient, viscosity, and density has not yet been developed and is beyond the scope of this work.

To make this point clear, we decided to modify the relevant text as follows:

"More importantly, the domain ~~expectedly~~ attached sooner in case of smaller damping ~~(smaller viscosity)~~, as observed in experiments for the domains on liquid copper as compared to liquid gold (Fig. 2(b)). This behavior suggests that the observed domain dynamics including the critical distance for attachment of the domains depend on material parameters other than the viscosity of the molten metal. Further detailed modelling is necessary to elucidate the issue."

Further, in the paragraph regarding the validity of the model, we modified following sentence: "Quantitative description of the observed phenomena requires detailed knowledge of the relation(s) between damping constants, viscosities and densities of the molten material,…"

Finally, in conclusions, we have changed the last sentence:

"Our experimental and modeling data reveal that ~~higher viscosity liquids~~ the choice of appropriate liquid substrate material facilitates stable oscillations of domains over longer periods of time and hence ~~may be desirable~~ is critical for achieving self-assembly and seamless stitching of domains, essential for large-area rheotaxy of single-crystalline sheets of 2D layers."

5. **Since the proposed model neglects electric dipole interactions - which may become significant for larger domains - the authors should briefly discuss how including these effects might alter their predictions.**

We thank the referee for the suggestion. Fig. 2c shows increased domain wobbling during the attachment/coalescence phase, an artefact of our current model, that is not observed in the experiments. We believe that adding electrostatic forces into the model would fix this issue.And, as mentioned in the manuscript and in the SI, our calculations of

the electrostatic forces suggest that these would be significant at much larger domain sizes than observed here which should increase the validity of modelling also to large scale domains.

In order to clearly communicate this, we have amended the text part where we discuss the validity of our model:

"We note that the model ~~neglects~~ does not include the influence of electric dipole interactions, which increase the repulsive interaction between domains and become more prominent with increasing domain size."
"We expect that the dipole-dipole interactions may be significant at the coalescence stage, contributing to the stabilization of domains before attachment. Therefore, accounting for electrostatic forces could potentially explain the difference between the model simulations (Fig. 2c) and our experiments (Fig. 2b) in the coalescence stage."

Reviewer: 2

Recommendation: This paper may be publishable, but major revision is needed; I would like to be invited to review any future revision.

Comments:

The article entitled 'Capillary wave driven dynamics of graphene domains during growth on molten metals' directly observes the translational, rotational, and oscillatory dynamics of graphene domains on a molten metal (Au/Cu) surface using in situ SEM, experimentally confirming the physical mechanism of capillary wave-driven selfassembly of two-dimensional materials. Furthermore, a continuum model is developed to couple capillary wave fluctuations with domain dynamics, successfully predicting oscillation, rotation, and critical adhesion behaviors. This paper provides a new paradigm for rheoepitaxy. However, the authors need to address the following issues before considering publication.

We thank the referee for recognizing the importance of our work and for taking time to critically review our work.

1. **The authors claim that capillary waves (induced by thermal fluctuations) are the physical essence of self-assembly of graphene domains on molten metals. How can the influence of thermal gradient forces/Marangoni effects be ruled out? What about short-range electrostatics? In short, how to verify that capillary waves are the dominant factor?**

We thank the referee for raising this issue. All of our experiments are carried out using over small (10s of micrometers in size) and large (mm-size) droplets that form by melting of solid chunks of the metals placed on the MEMS heating chips or the heating wire, respectively. Both effects mentioned by the referee (thermal gradient and Marangoni effects) would significantly and differently affect the domain behavior on gold and copper due to different substrate scaling and composition (difference in vapor pressure and, hence, substrate evaporation rate). Our experiments show that this is not the case, making a strong argument to rule these and similar effects out. To make this point clear, we have added the following sentence in our discussion section:

"Given that all our experiments are carried out on fairly large (tens of micrometers up to millimeter size) metal droplets and since all our detailed in situ observations are limited to fields of view much smaller than the substrate size, we expect that the influence of thermal gradients, if any, on graphene domain dynamics is insignificant. "

Regarding the influence of other factors, we have discussed the effects of vdW forces, electrostatic interactions, and capillary forces on the observed self-assembly in the SI. The experiments addressing the other possible explanations of wobbling are presented in Figs. S11S16.

Nevertheless, this discussion seems hidden in the SI. Therefore, to stress it in the main text, we have modified the following sentence:

"…that the distances between metastable floating domains are in nanometer range, which cannot be explained by electrostatic, van der Waals and capillary forces alone.[18,38,39] We provide additional discussion of the other interactions in SI."

To identify the dominant mechanism(s), we have carefully investigated the effects of different experimental conditions on the wobbling of the domains and their assembly.

We performed experiments on two completely different systems (liquid gold: substrate size of several tens of micrometers, high pressure conditions, graphene flakes of 250 nm to 5 □m, different heating system from that used for copper: millimeter-sized substrate, high vacuum, graphene flakes of 1 □m to 5 □m). Despite these differences, in both systems the behavior of graphene flakes is strikingly similar, even quantitatively, as visible on A(R-r) plot. We emphasize that this is the strongest argument in favor of our hypothesis.

As for (repulsive) electrostatic forces, we do not rule them out. However, we cannot implement them into the current model, because that requires quantitative knowledge of properties of the capillary waves. Such data is missing and experimentally beyond our

current capabilities. Nevertheless, we note that the electrostatic forces alone cannot explain several experimental observations (e.g. attraction of the domains) and there is no other attractive interaction that, paired with the electrostatic one, would explain the experimentally observed behavior. On contrary, the capillary waves-based model qualitatively explains all the experiments. We believe that adding electrostatic forces into the model would fix e.g. increased domain wobbling during the attachment phase, which is observed in the model but largely suppressed in the experiment. In addition, our calculations of the electrostatic forces suggest that these would be significant at much larger domain sizes than observed here.

In order to clearly communicate this issue, we have amended the text part where we discuss the validity of our model:

"We note that the model ~~neglects~~ does not include the influence of electric dipole interactions, which increase the repulsive interaction between domains and become more prominent with increasing domain size."
"We expect that the dipole-dipole interactions may be significant at the coalescence stage, contributing to the stabilization of domains before attachment. Therefore, accounting for electrostatic forces could potentially explain the difference between the model simulations (Fig. 2c) and our experiments (Fig. 2b) in the coalescence stage."

2. In the AFM image in Figure 1e, why is the height of graphene is 3 nm, while the typical monolayer thickness is 0.34 nm?

We agree! We attribute this result to limitations of using AFM at high-temperatures (> 1000 °C) for measuring step heights on liquid substrates. As the referee may be aware, the topic of graphene step edge height has been widely discussed since the discovery of graphene. For example, in [https://doi.org/10.1021/nl061420a] they calculated the height of multilayer graphene h as h = n t + t0; where n is the number of layers, t = 0.33 nm is approximately equal to the interlayer distance in graphite and t0 = 0.35 nm is an „instrumental offset“. In [https://doi.org/10.1016/j.carbon.2008.06.022] the phenomenon is explained more thoroughly and it is claimed that unless special care is taken, the step heights obtained can vary significantly among images. In line with these studies, we have observed that changing the setpoint of our measurement gives different graphene step heights.

We would like to point out that our measurements are even more complicated as the graphene is localized on a liquid. In contrast to graphene on solid substrate, the graphene-liquid surface is less rigid; in addition, the tip may get immersed into the liquid to a certain extent while at the lowest oscillation point. Hence, we do not emphasize the step height

measurements in the manuscript; instead we limit the conclusions from AFM measurements to the fact that a large meniscus around the graphene domains is absent.

To avoid confusion, we have added following text to the SI (Fig. S10, caption): "Note that the measured step height is dependent on the instrumental settings

[https://doi.org/10.1016/j.carbon.2008.06.022] and, therefore, not realistic. The AFM measurement is thus used solely to dismiss the possible meniscus presence."

3. **Figure 2b shows that amplitude (A) decays nonlinearly with (R-r), but why do the data for domains of different sizes almost overlap? Does the amplitude (A) depend only on the domain distance (R-r)?**

That is correct! The domain oscillation amplitude (A) vs. the inter-domain distance (R-r) does not depend solely on the domain size. This size-independence has been the reason for introducing the model based on capillary waves – a purely geometrical, scaling-free effect. We have not found any correlations between the amplitude of wobbling and other system variable (e.g. if A is plotted against r or R) that would be independent of (R-r). Only A(R-r) plot shows a clear, reproducible trend, which indicates that the amplitude is dominated by the distance between the flakes.

To make this point clear, we have revised the relevant part of the text and amended the rest:

"Importantly, ~~A scales only with the available free liquid space, i.e. (R-r), and is independent of r, shape, composition of the liquid catalyst (i.e. Au or Cu) (see Fig. 2(b)), ethylene partial pressure (i.e. deposition flux), experimental setup and imaging procedure (i. e. beam-induced effects). Detailed discussion of these observations is provided in SM. A~~all the data obtained from domains of different sizes, shapes, in different environments, on Cu and Au collapse onto a single curve (Fig. 2b). Further detailed discussion of additional experimental observations is provided in SI."

4. **Figure 2b shows that the experimentally observed the critical distance of gold (50 ± 20 nm) is less than that of copper (140 ± 60 nm). Stokes' law predicts that highviscosity media have greater damping and domain motion should stop earlier, and gold with its higher viscosity (Described in the article as 1.35× larger), should have a larger critical distance. Please explain.**

We thank the referee for this note. We partially agree with the reasoning above. But the system we have studied is different: the wobbling domain represents a <u>driven</u> oscillator. Hence, the domain movement is continuously damped by a force whose magnitude is to some extent proportional to the liquid viscosity, as correctly stated by the referee. However,

at the same time, the movement is driven by the capillary waves, which exert a force on the flake whose magnitude is also dependent on the liquid viscosity.

The critical distance represents a moment when the forces that push the domain from different sides are all equal. This quasi equilibrium is quickly broken because the domain grows in time, and the domains coalesce because the forces on one side prevail. The model outcomes (in agreement with experiment) suggest that this quasi-equilibrium is reached sooner on substrates with smaller damping, which we relate to lower viscosity.

As indicated in our response to Referee #1's comment #4, the explanation presented in the manuscript ("More importantly, the domain expectedly attached sooner in case of smaller damping (smaller viscosity),....") is not entirely correct because of role of other material parameters such as liquid substrate density on the domain dynamics. We have therefore modified the relevant text as follows:

"More importantly, the domain ~~expectedly~~ attached sooner in case of smaller damping ~~(smaller viscosity)~~, as observed in experiments for the domains on liquid copper as compared to liquid gold (Fig. 2(b)). This behavior suggests that the observed domain dynamics including the critical distance for attachment of the domains depend also on material parameters other than the viscosity of the molten metal. Further detailed modelling is necessary to elucidate the issue."

Further, in the paragraph regarding the validity of the model, we modified following sentence: "Quantitative description of the observed phenomena requires detailed knowledge of the relation(s) between damping constants, viscosities and densities of the molten material,…"
Finally, in conclusions, we have changed the last sentence:

"Our experimental and modeling data reveal that ~~higher viscosity liquids~~ the choice of appropriate liquid substrate material facilitates stable oscillations of domains over longer periods of time and hence ~~may be desirable~~ is critical for achieving self-assembly and seamless stitching of domains, essential for large-area rheotaxy of single-crystalline sheets of 2D layers."

5. In Figure 3a, the domains rotate while growing from a circular to a hexagonal shape. Is the reorientation of domains related to the electrostatic forces on the carbon atoms at the edge of the sample (electrostatic torque)?

This is indeed very good point. The capillary waves-based model and electrostatic calculation yield the same result – both mechanisms enforce the hexagonal flakes rotate until their edges are parallel. In capillary waves-based model, this is due to anisotropy in wavevectors that fit in between the domains if not parallel. This effect is visible in our movies, which show changes in capillary waves' amplitudes across the edges if they are

misoriented. The model based solely on the capillary waves is able to explain both the rotation and alignment.

To make this point clear, we have changed the following sentence in the section where we discuss the validity of our model. We deleted "~~Our model's predictions are in qualitative agreement with the experimental observations of domain oscillations and rotations (see Figs. 2 and 3), specifically that the domain position depends only on the distance between the adjacent domains (Fig. 2b).~~" and instead, inserted "The observed reorientation of domains during growth can be attributed to the operation of capillary waves and electrostatic forces. We note that our model based solely on the capillary waves is able to explain both the alignment (Fig. 2) and rotation (Fig. 3) observed in experiment; domain rotation occurs due to anisotropy in wavevectors that fit in between the domains that are not parallel. This effect is visible in our movies, which show changes in amplitudes of capillary waves across the edges of misoriented domains. "

The model assumes perfect hexagons (Figure 3b), but the experimental domain edges are irregular (jagged in Figure 3a). Does the actual edge roughness significantly alter the capillary wave force field distribution? Is hexagonal symmetry the key and growth process dominated?

As noted by the referee, any irregularity of the flake shape from perfect hexagon may affect the rotation and domain alignment with respect to each other. Perfect hexagonal shape (or any other regular shape that is observed in 2D materials, e.g. triangular) is a necessary requirement for perfect self-assembly and alignment.

In response to this note, we have added the following text at the end of the section dealing with domain rotation:

"However, note that the model assumes perfectly smooth domain edges. A significant edge roughness, as sometimes observed in experiment, may prevent the parallel positioning of the domain edges."

6. Does high-energy electron beam irradiation induce local thermal gradient? Electron beam perturbations (e.g., low beam current contrast) or thermal gradient effects have not been ruled out. How to reduce the influence of electron beam voltage and current in experiments?

Referee raises a valid concern regarding the role of electron beam irradiation on the observed phenomena. We carried out a series of experiments aimed at identifying the role of electron beam on the domain dynamics. The results are presented in the SI, based on

which we rule out the electron beam effects (including heating) on the domain growth kinetics, wobbling amplitudes, and reorientation dynamics.

In Fig. S13, we have focused primarily on the possibility whether the wobbling is induced by the electron beam. The wobbling occurs also on previously unexposed part of the sample, even if imaged by a much smaller electron dose (because of changing magnification). The images Fig. S15 suggest that the amplitude of wobbling is not affected by the beam (up to the resolution of these images), as we can reconstruct similar domain trajectory under different beam conditions (we have changed dwell time).

The argument of local heating by the beam is weakened by the experiments summarized in Fig. S15, as they are performed with different beam flux, yet with similar results. Additionally, local heating would cause thermomigration of the floating domains to or from the beam – we have never observed such behavior. Also, we did not observe changes in graphene growth rates with and without beam exposure. To further support our argumentation, we note that the beam currents used in this work varied between 50 pA and 1 nA, which is significantly below beam currents that can induce localized sample heating (an estimate is provided e.g. here https://doi.org/10.1016/j.ultramic.2025.114195), especially on metallic sample.

To stress this important part of our work, we have added following sentence to the main text: "We have performed several control experiments to assess the possible effect of the electron beam (momentum or charge transfer, local heating etc.) on the observed phenomena, concluding that the beam effects are negligible (see Supporting Information for further details)."
In addition, we have modified the relevant section in SI as follows:

"SEM images are acquired in the secondary electron mode using 1~~0~~-20 kV accelerating voltages and electron beam currents between 50 pA and up to 1 nA to minimize charging and thermal gradients possibly caused by the electron-beam."
"Additional experiments (Fig. S15) demonstrate that the trajectories of the domains deduced from image sequences taken under different beam conditions remain similar."
jz-2025-023212.R2

Name: Peer Review Information for "Capillary wave driven dynamics of graphene domains during growth on molten metals"

Second Round of Reviewer Comments

Reviewer: 2

Comments to the Author

The authors have provided reasonable explanations and detailed discussions for most of my questions, supplemented with some additional supporting data and analysis. The paper's conclusions remain convincing, but the capillary wave interpretation need further discussion. Therefore, the authors need to make some minor revisions to the current version. I still have some concern.

1. Capillary waves are essentially a random, statistical physical process. On the surface of molten copper, capillary waves reflect, superimpose, and form standing wave patterns. How does this "random" process achieve the "ordered" arrangement of a large number of parallel graphene edges? What is the distribution of capillary waves on the molten metal surface? This has implications for guiding the growth of large-scale single-crystal graphene on molten copper surfaces.

2. What is the spacing (or gap) between molten copper (or gold) and graphene? Is it an atomic-level interfacial spacing? Is it a spatially non-uniform dynamic variable (from center to edge)? This gap value is crucial for capillary wave forces to effectively drive the motion of graphene domains.

Author's Response to Peer Review Comments:

Reply to reviewers

Below please find point-to-point response to the reviewers' comments. For clarity, we used **bold font** for the referee comments and italics for our responses, blue color for the revisions/new text, ~~crossed~~ for deleted text.

Reviewer: 2

Recommendation: This paper is publishable subject to minor revisions noted. Further review is not needed.

We sincerely thank the reviewer for a second review of our manuscript and appreciate the referee's recommendation to publish without further review.

The authors have provided reasonable explanations and detailed discussions for most of my questions, supplemented with some additional supporting data and analysis. The paper's conclusions remain convincing, but the capillary wave interpretation need further

discussion. Therefore, the authors need to make some minor revisions to the current version. I still have some concern.

1. Capillary waves are essentially a random, statistical physical process. On the surface of molten copper, capillary waves reflect, superimpose, and form standing wave patterns. How does this "random" process achieve the "ordered" arrangement of a large number of parallel graphene edges? What is the distribution of capillary waves on the molten metal surface? This has implications for guiding the growth of large-scale singlecrystal graphene on molten copper surfaces.

We agree, capillary waves have truly stochastic behavior. However, some of their characteristics have to obey the conditions that are given by the system.

Firstly, the boundary conditions rising from instant positions of all edges of the graphene domains select which standing wave (in fact, particular solution of the Helmholtz equation) is present. Since the graphene domains are permanently moving, these conditions are temporarily changing and, therefore, different standing waves are excited at different instants. This indicates that some specific orientations of the domain edges could be preferred despite the random essence of the capillary waves. In other words: As the referee correctly points out, due to a specific selection of generally accidental capillary waves, even large-scale graphene crystals are guided to certain directions and orientations leading to an ordered arrangement.

Secondly, since the capillary waves demonstrate a periodic mechanical motion, they are also characterized by frequency. Thus, the model assumes that their typical amplitude is not random but rather follows a time-independent dispersion curve similarly to the case of a driven harmonic oscillator. This assumption has been experimentally verified and this reference is given in the model description in SI ("The dispersion curve [doi.org/10.1017/jfm.2021.164] results from the following consideration: the liquid behaves as a resonant system exhibiting a certain resonant frequency."). Put altogether, the following physical concept arises: There are waves with particular wavelength having largest amplitude, while waves with lower or higher wavelengths show significantly lower amplitudes. Such wave distribution introduces both repulsive- and attractive forces into our model: At large distances between two domains the capillary waves in between push them "repulsively" because they have larger amplitudes than the outer ones. On the other hand, at low distances capillary waves in between vanish and, therefore, the outer waves push them "attractively". This phenomenon is well-known in quantum electrodynamics as the Casimir effect or Casimir force, although the acting forces have unrelated origin.

In conclusion, although capillary waves represent a stochastic process, both the boundary conditions and the resonant curve have direct influence on their distribution on the surface as described. The images resulting from our 2D modelling capture the force amplitudes acting on the graphene domains. We could potentially show such images for many different wavelengths that act on the flake, but these datasets are quite large and we believe not necessary.

Nevertheless, the core message in reviewer's question is important to address. We have added following text to the part where we discuss validity of our model:

"The observed reorientation of domains during growth can be attributed to the operation of capillary waves and electrostatic forces. Although the capillary waves arise from a stochastic process, the boundary conditions posed by the graphene domain edges selectively restrict certain wavelengths of the generated standing waves. ~~We note that o~~Our model based solely on the capillary waves thus ~~is able to~~ explains both the alignment (Fig. 2) and rotation (Fig. 3) observed in experiment despite the stochastic nature of the capillary waves;..."

2. What is the spacing (or gap) between molten copper (or gold) and graphene? Is it an atomic-level interfacial spacing? Is it a spatially non-uniform dynamic variable (from center to edge)? This gap value is crucial for capillary wave forces to effectively drive the motion of graphene domains.

We agree with the reviewer that the gap size determination is important in our argumentation towards the capillary wave-driven graphene domain mechanics. As we have discussed previously, such information is rather challenging to deduce from our HT AFM data. Nevertheless, we can reference experimental data acquired by X-Ray Reflectivity on graphene/molten copper system, as well as associated theoretical modelling (10.1002/advs.202204684, 10.1107/S1600577522002053, 10.1002/admi.202300053, 10.1021/acsnano.0c10377), reporting a gap in the range <1.5 – 3> Å. This range is comparable to "roughness" induced by capillary waves on the liquid metal surface. Importantly, a different "roughness" was measured on liquid copper and graphene floating on its surface (10.1107/S1600577522002053), suggesting substantial interaction between capillary waves and graphene. Such data support our hypothesis of capillary waves-driven domain motion and self-assembly. In order to improve and support our argumentation, as proposed by the reviewer, we have made following amendments of the text:

"The related deformation of the liquid surface exerts a mechanical force on the floating object, making it move and rotate, as has been demonstrated for colloidal microparticles.[36,38] Given that the surface undulations induce surface roughness (1.5 to 3 Å) comparable to the gap between the floating graphene domain on liquid surface [10.1002/advs.202204684,

10.1107/S1600577522002053, 10.1002/admi.202300053, 10.1021/acsnano.0c10377], we assume similar interaction between surface undulations and graphene [10.1107/S1600577522002053]."

Regarding the uniformity of this gap across the graphene domains, it is important to note that at the high temperatures used in our experiments, adsorbate-terminated graphene domain edges become unstable and terminating atoms/molecules are preferentially desorbed. Consequently, metal-terminated edges are favored instead of adsorbate-terminated ones [10.1021/ja405499x, 10.1021/acs.jpclett.5b01841]. As a result, the domain edges are likely bent towards the liquid metal surface, as suggested by theoretical modelling referred above (similarly to our DFT modelling presented in Fig. S4), which further support the likelihood of capillary wave interaction with graphene edges.

However, direct experimental verification of graphene edge bending in our experimental conditions is challenging. The bending occurs at the atomic scale, meaning that techniques such as scanning tunneling microscopy would be required. In principle, our SE images may also reveal this close-to-edge bending, but the lateral resolution of our images is insufficient to conclusively confirm or disprove the effect, despite careful examination of the data. We show these data in the new Fig. S9, and we have added a note to the SI (Discussion of possible meniscus):

"It should be noted that neither HT-AFM nor SEM images acquired during our experiments allow the detection of nanoscale surface undulations of the liquid metal surface or possible Ångstrom-scale bending at the graphene edge predicted by the DFT calculations, due to limited lateral resolution."

New Fig. S9:

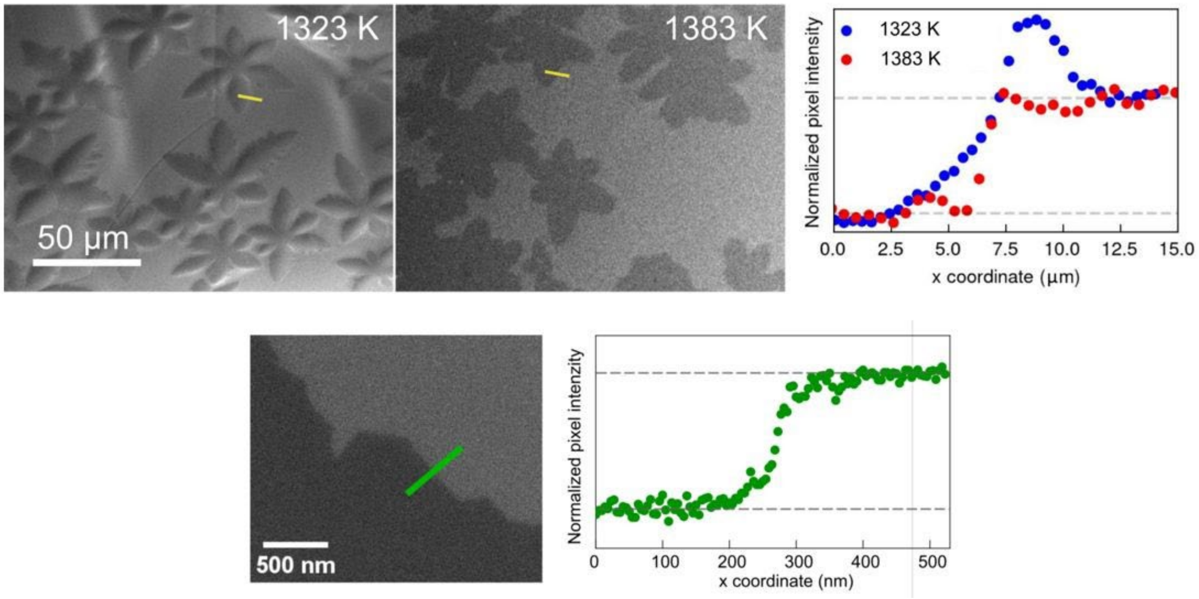

Figure S9. Representative secondary electron (SE) images of graphene domains grown on pre-molten and liquid Cu at different temperatures, and on liquid Au. First, we note that SEM images do not show any ‘rim’ around the graphene domain edges on solid ~~Cu~~ substrates (Fig. 1). (top) In this experiment, graphene was grown with pC2H4 = 1 ×10-2 Pa at T = 1323 K, slightly below the melting point Tm, Cu of Cu, a bright rim appears around the domains. The rim disappears when the substrate is molten at T = 1383 K. Generally, secondary electrons are very sensitive to the curvature of the emitting surface (observation of single atomic steps is possible)18, hence, any nanometer-scale irregularity of the surface is enhanced in SE image. The SE signal profiles on the right, measured along the yellow lines in the SEM images, demonstrate SE signal enhancement around the domain’s edges just before melting, indicating surface swelling around the domain. Upon melting, the surface becomes flatter and the SE signal enhancement disappears. This observation supports the conclusion that no large-scale liquid meniscus forms around the graphene domains on a fully molten substrate. Electron beam imaging conditions: 5 keV, 1 nA. (bottom) Similar to Cu, graphene domain grown on liquid gold (pC2H4 = 30 Pa at T = 1363 K) does not exhibit any meniscus. Electron beam imaging conditions: 10 keV, 200 pA.
